# Converging GWAS and Genome Editing Decrypts MsCBF8‐ *MsGolS2* Regulatory Module for Drought Tolerance Without Yield Penalty in *Medicago sativa*

**DOI:** 10.1111/pbi.70756

**Published:** 2026-09-22

**Authors:** Yangyang Zhang, Xueqian Jiang, Tian Zhang, Huajuan Leng, Jing Cui, Xu Jiang, Lili Zhang, Zhen Wang, Tiejun Zhang, Qingchuan Yang, Xue Wang, Junmei Kang

**Affiliations:** ^1^ Institute of Animal Science Chinese Academy of Agricultural Sciences Beijing China; ^2^ Department of Agronomy and Horticulture University of Nebraska‐Lincoln Lincoln Nebraska USA

**Keywords:** alfalfa, drought, MsCBF8‐*MsGolS2*, stomata

## Abstract

Drought constitutes a major abiotic stress limiting alfalfa productivity. To elucidate the genetic basis of drought tolerance, a genome‐wide association study (GWAS) was conducted in alfalfa, which identified a CBF/DREB1 family transcription factor designated MsCBF8. Utilizing an optimized CRISPR‐Cas9 system, mutants were successfully generated in the complex autotetraploid background. Combined with overexpression and Arabidopsis complementation assays, this confirmed that MsCBF8 functions as a positive regulator of drought tolerance, with its overexpression enhancing drought tolerance without compromising plant growth. Physiological analyses revealed that MsCBF8 improves drought tolerance by coordinating antioxidant defence (elevated SOD and CAT activities, decreased H_2_O_2_ and MDA contents), dynamically regulating stomatal movement (increased water‐use efficiency), and maintaining photosynthetic performance. At the molecular level, MsCBF8 directly and specifically binds to the GCCGAC *cis*‐element in the *MsGolS2* promoter and activates its expression. Silencing *MsGolS2* via virus‐induced gene silencing (VIGS) reduced drought tolerance and aggravated oxidative damage, providing the first functional evidence for the essential role of this gene in the drought response of alfalfa. This study systematically elucidates a novel MsCBF8‐*MsGolS2* regulatory module, offering crucial insights into the drought adaptation mechanisms in alfalfa and providing valuable genetic resources and breeding targets for developing high‐yield, water‐efficient and drought‐tolerant alfalfa cultivars.

## 1 Introduction

Under the escalating global warming, drought has emerged as the most severe abiotic stress factor constraining agricultural productivity (Sun et al. 2022; Sato et al. 2024). Over the past decade, drought‐caused crop yield losses have exceeded $30 billion annually worldwide, posing a substantial threat to global food security (Gupta et al. 2020). In this context, elucidating the molecular mechanisms underlying plant drought tolerance and developing novel high‐yield, drought‐tolerant and water‐use‐efficient crop varieties are crucial for ensuring sustainable agricultural development. To combat drought stress, plants employ multi‐layered adaptive strategies including morphological remodelling (e.g., root system architecture modification), physiological regulation (including stomatal aperture regulation, osmotic homeostasis maintenance and redox equilibrium preservation) and molecular coordination (such as activation of stress‐responsive genes encoding LEA proteins and aquaporins) (Gupta et al. 2020). These sophisticated drought adaptation mechanisms are orchestrated by pivotal transcription factors, including AREB/ABF, NAC, MYC, WRKY and DREB2 families (Kim et al. 2024), which function as central regulators by integrating upstream signalling cascades and initiating downstream gene expression networks in plant drought tolerance.

As central components of the DREB transcription factor family, CBF/DREB1 (C‐repeat binding factor/DRE‐binding protein 1) and DREB2 play pivotal regulatory roles in plant abiotic stress responses through specific binding to DRE *cis*‐acting elements to activate the expression of downstream stress‐responsive genes (Zhang and Xia 2023). While DREB2 primarily mediates drought, salinity and heat stress responses (Razi and Muneer 2021; Sato et al. 2024), CBF/DREB1 was initially characterized as the master regulator of cold signalling pathways (Kidokoro et al. 2022). Emerging evidence, however, reveals the involvement of CBF/DREB1 in drought adaptation. For instance, transgenic wheat ( *Triticum aestivum* ) expressing soybean ( *Glycine max* ) *GmDREB1* exhibited 4.79%–18.43% grain yield enhancement under field drought conditions with improved drought tolerance (Zhou et al. 2020). Similarly, *Musa nana* overexpressing *MaDREB1F* demonstrated synergistic drought tolerance through three‐pronged mechanisms: enhanced accumulation of osmoregulatory substances, reinforced cellular antioxidant defence systems and activation of jasmonic acid/ethylene signalling pathways (Xu et al. 2022). Cutting‐edge studies further unveil that CBF/DREB1 and DREB2 can form regulatory cascades to synergistically amplify plant drought resilience (Wei et al. 2023). These findings collectively position CBF/DREB1 not merely as an independent drought‐responsive hub, but rather as an integrative node capable of coordinating multiple stress signalling networks, thereby offering an efficient genetic target for crop stress tolerance improvement.

As a globally cultivated high‐quality forage crop, alfalfa ( *Medicago sativa* ) is prized for its elevated protein content and robust stress tolerance, playing a pivotal role in the livestock industry and ecological restoration (He, Chen, et al. 2025). The vegetative growth stage of this crop exhibits exceptional sensitivity to drought stress, triggering adaptive responses such as growth reduction and premature flowering that ultimately compromise yield and forage quality, significantly constraining alfalfa industry development (Yang et al. 2025). This underscores the critical importance of molecular breeding strategies employing transgenic and gene‐editing technologies to develop high‐yield, drought‐tolerant alfalfa cultivars. Recent advancements in drought tolerance mechanisms have identified multiple functional genes providing theoretical foundations for breeding drought‐tolerant cultivars. For example, *MsCYP71*, *MsNTF2L* and *MsMYBH* enhance drought tolerance through mechanisms involving antioxidant defence potentiation, photosynthetic efficiency preservation and stomatal dynamics optimization (Luo et al. 2022; Liu, Shi, et al. 2023; Shi, Liu, et al. 2024). Moreover, heterologous expression of *MtNAC33* has been demonstrated to significantly improve alfalfa drought tolerance (Yang et al. 2025). Notably, despite whole‐genome analyses identifying 25 *CBF*/*DREB1* family genes in alfalfa (Cui et al. 2025), functional characterization of this transcription factor family in drought responses remains markedly understudied compared to model plants. The inherent complexity of alfalfa autotetraploid genome presents substantial technical barriers, particularly in genetic manipulation, where the application of genome editing technologies for functional characterization remains particularly challenging and underutilized. Current evidence has primarily validated limited family members involved in cold stress responses, while the drought‐responsive regulatory networks mediated by CBF/DREB1 transcription factors await systematic elucidation.

Genome‐wide association study (GWAS) has emerged as a pivotal strategy for deciphering genetic determinants underlying complex traits in plants. To elucidate the genetic basis of drought tolerance in alfalfa, we initially employed *k*‐mer‐based GWAS (Voichek and Weigel 2020; He et al. 2024) for gene mining. This study demonstrates that *k*‐mer‐based GWAS serves as an effective strategy for mining drought‐tolerance genes in alfalfa. Through this approach, we identified a CBF/DREB1 family homologue, designated MsCBF8. To elucidate its function and mechanism in drought response, we integrated genome editing, overexpression, complementation assays and physiological‐biochemical analyses to systematically validate the key role of MsCBF8 in drought tolerance. Furthermore, we revealed a molecular module through which MsCBF8 enhances drought tolerance by directly and specifically regulating *MsGolS2*, a key gene in the raffinose biosynthesis pathway. This work provides novel genetic resources and a theoretical foundation for elucidating the regulatory network underlying drought adaptation in alfalfa.

## 2 Results

### 2.1 MsCBF8 Functions as a Drought‐Responsive Transcription Factor

**FIGURE 1:**
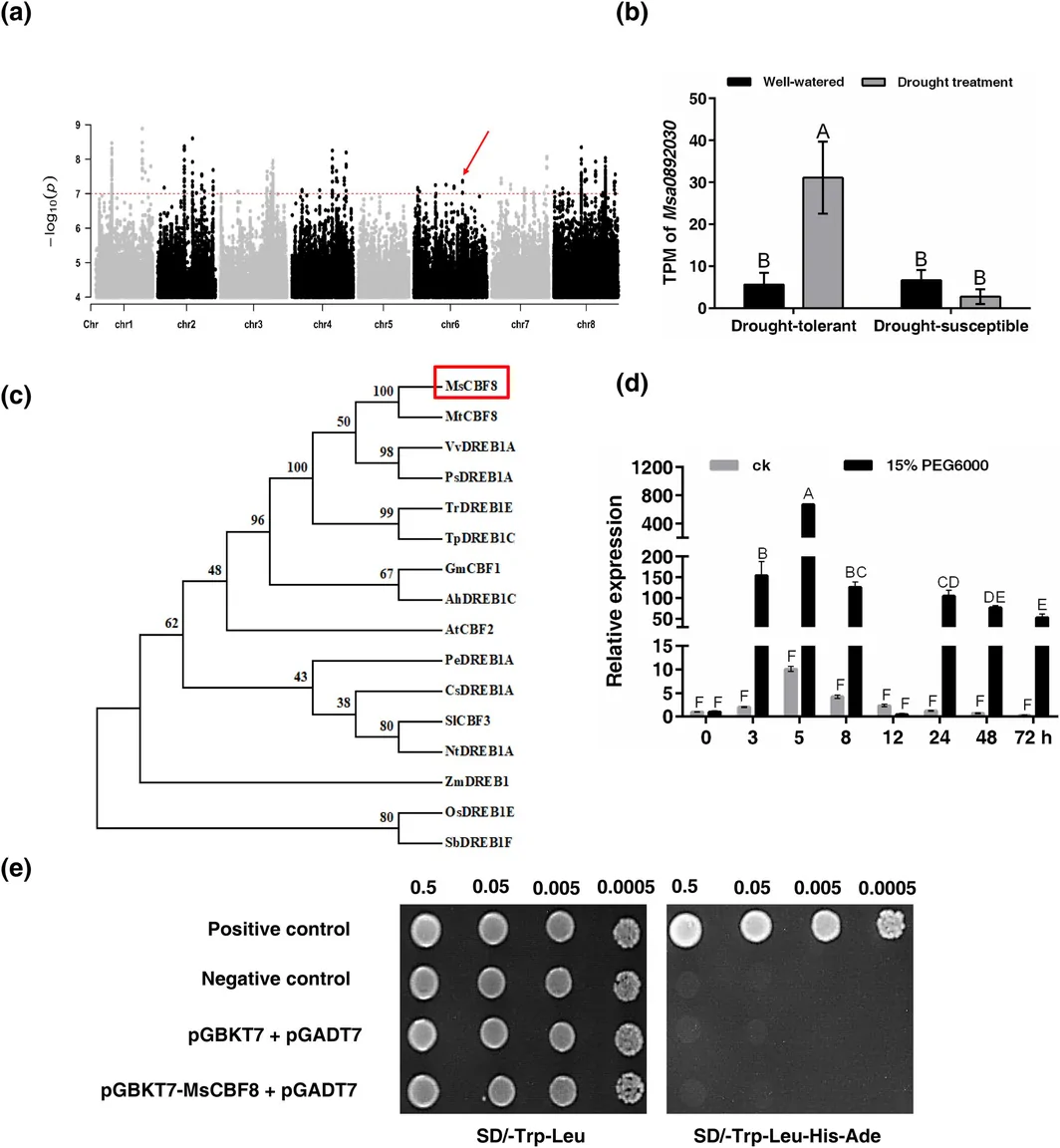
MsCBF8 is a drought‐responsive transcription factor. (a) Manhattan plots of GWAS for drought‐related phenotype (SLR‐DRV). (b) TPM of *Msa0892030* in drought‐tolerant genotype (PI 641381) and drought‐susceptible genotype (Beaver) under well‐watered and drought conditions. (c) Phylogenetic analysis of MsCBF8 with CBF/DREB1 from *Medicago truncatula* (MtCBF8), soybean ( *Glycine max* , GmCBF1), peanut ( *Arachis hypogaea* , AhDREB1C), pea ( *Pisum sativum* , PsDREB1A), red clover ( *Trifolium pratense* , TpDREB1C), white clover ( *Trifolium repens* , TrDREB1E), *Arabidopsis thaliana* (AtCBF2), maize ( *Zea mays* , ZmDREB1), rice ( *Oryza sativa* , OsDREB1E), poplar (*Populus euphratica*, PeDREB1A), tomato ( *Solanum lycopersicum* , SlCBF3), cucumber ( *Cucumis sativus* , CsDREB1A), tobacco ( *Nicotiana tabacum* , NtDREB1A), grapevine ( *Vitis vinifera* , VvDREB1A), and sorghum ( *Sorghum bicolor* , SbDREB1F). (d) *MsCBF8* expression pattern in leaves of 4‐week‐old hydroponically cultured alfalfa subjected to 15% PEG6000. (e) Autonomous transactivation assay of MsCBF8. The growth state of co‐transformants with BD‐MsCBF8 and pGADT7 (AD)‐fusion constructs were examined in SD/−Trp‐Leu and SD/−Trp‐Leu‐His‐Ade medium. Error bars represent the mean ± standard error of the mean (SEM) from three biological replicates. Statistical significance between groups was determined by one‐way analysis of variance (ANOVA). Bars labelled with different uppercase letters indicate statistically significant differences at *p* < 0.05 based on Duncan's multiple range test.

A GWAS identified a significant locus at 69.52 Mb on chromosome 6 (kmer_chr6_69525041) (Figure 1a and Table S2), a region adjacent to a previously reported fall dormancy‐associated signal (chr6_69737395) (Zhang, Kang, et al. 2023), suggesting its potential role in stress regulation. Within this locus, we pinpointed *Msa0892030*, a gene that shows stronger drought‐induced up‐regulation in a drought‐tolerant genotype (PI 641381) than in a susceptible one (Beaver) (Singer et al. 2021) (Figure 1b). Phylogenetic analysis demonstrated a closest evolutionary relationship between this protein and MtCBF8 from *M. truncatula* , leading to its designation as MsCBF8 in alfalfa (Figure 1c). The gene encodes a 279 amino‐acid protein (32.0 kDa, pI 9.26) that lacks a signal peptide, is rich in random coils (62.01%), and contains a canonical AP2 domain (residues 54–112) (Figure S1). Tissue‐specific expression profiling indicated ubiquitous expression of *MsCBF8* in roots, stems and leaves, with significantly higher transcript levels in roots and stems than in leaves (Figure S2). Under 15% PEG6000‐induced drought, *MsCBF8* displayed a dynamic expression pattern: a rapid 77‐fold induction at 3 h post‐treatment, followed by a decrease to baseline by 12 h, and maintained up‐regulation during extended stress (Figure 1d). Yeast two‐hybrid assays confirmed that MsCBF8 lacks autonomous transactivation activity, as co‐transformants carrying MsCBF8‐pGBKT7 (BD) and pGADT7 (AD) plasmids failed to grow on selective SD/−Trp‐Leu‐His‐Ade medium, similar to negative and empty‐vector controls (Figure 1e).

### 2.2 Overexpression of *MsCBF8* Enhances Drought Tolerance in Alfalfa

**FIGURE 2:**
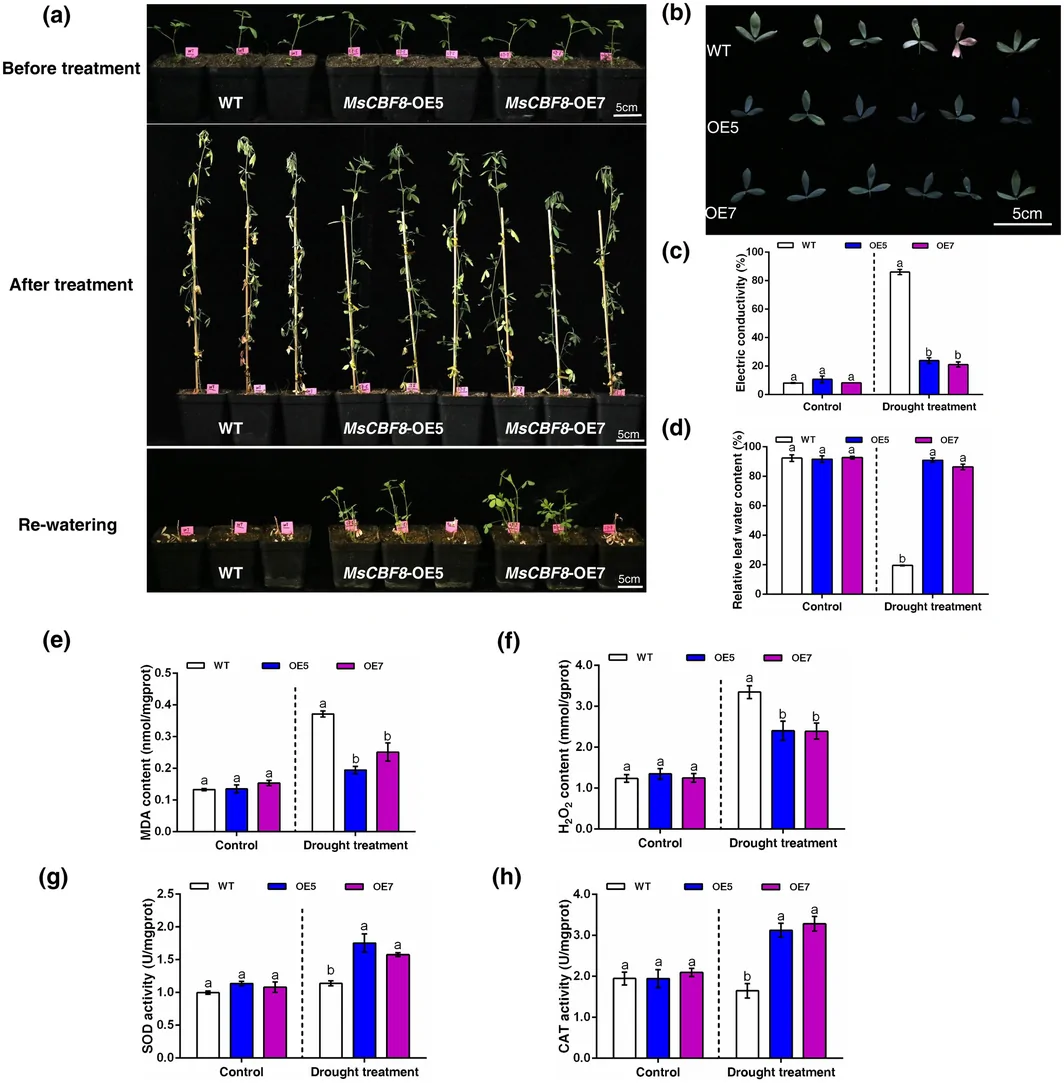
*MsCBF8*‐overexpressing alfalfa exhibited enhanced drought tolerance. (a) The growth performance of *MsCBF8*‐overexpressing (*MsCBF8*‐OE) and wild type (WT) alfalfa plants before treatment, after natural drought treatment, and re‐watering. Uniform cuttings for 25 days were transplanted into plastic pots (8.5 × 8.5 × 9.5 cm) containing growth substrate (vermiculite: Nutrient soil = 2:1). Prior to transplantation, all pots with growth substrate were preconditioned in a standardized tray (28.95 × 28.95 × 5.3 cm) containing ~2 L water overnight to achieve complete saturation of each pot. Following overnight saturation, individual plants were transplanted with excess water removed from the tray. No more watering during seedling growth, waiting for the natural drought for 1 month to begin to observe and record the phenotype. After pruning, the phenotype was observed after rehydration for 1 week. (b) Leaf growth state of *MsCBF8*‐OE and WT plants after drought treatment. (c–h) Electric conductivity, relative leaf water content, MDA, H_2_O_2_ content, SOD and CAT activity of *MsCBF8*‐OE and WT after drought treatment. Error bars represent the mean ± standard error of the mean (SEM) from three biological replicates. Statistical significance between groups was determined by one‐way analysis of variance (ANOVA). Bars labelled with different lowercase letters indicate statistically significant differences at *p* < 0.05 based on Duncan's multiple range test.

To further investigate the biological function of *MsCBF8*, we generated overexpression lines (*MsCBF8*‐OE) in alfalfa through *Agrobacterium*‐mediated transformation in alfalfa, with two independent transgenic lines (OE5 and OE7) selected for subsequent analyses (Figure S3). Following 25 days of vegetative reproduction in vermiculite, uniformly developed wild‐type (WT) and *MsCBF8*‐OE plants were transplanted into soil, and the soil moisture was saturated before the drought was applied. After initial watering, all plants underwent complete water withdrawal for 30 days under controlled conditions until wild‐type (WT) plants exhibited wilting. After 30 days of natural drought treatment, WT plants exhibited severe wilting and chlorosis, whereas both OE lines maintained green leaf phenotypes with superior drought tolerance. Following drought treatment, plants were rewatered and allowed to recover for 7 days. The *MsCBF8*‐OE lines showed a significantly higher survival rate compared to the WT controls (Figure 2a,b and Figure S4). Physiological analyses demonstrated that under drought stress, the electric conductivity in OE lines was reduced by 62%–65% relative to WT (*p* < 0.05) (Figure 2c), while the relative water content in leaves reached 91% (OE5) and 86% (OE7), representing 67%–71% increases over WT levels (*p* < 0.05) (Figure 2d).

In addition, we also examined the changes of physiological indices related to oxidative stress and antioxidant response of plants after drought stress. Results showed that the malondialdehyde (MDA) content in OE lines decreased by 32%–48% relative to WT (*p* < 0.05), concomitant with a 28% reduction in H_2_O_2_ accumulation (Figure 2e,f). SOD and CAT activities in transgenic OE plants increased by 1.5‐ and 1.9‐fold, respectively, compared to drought‐stressed WT (Figure 2g,h). These multi‐layered evidences conclusively demonstrated that *MsCBF8* overexpression enhances drought tolerance through coordinated regulation of improved membrane stability, water retention capacity, oxidative stress mitigation and antioxidant defence activation in alfalfa.

### 2.3 *MsCBF8* Mutation Compromises Drought Tolerance in Alfalfa

**FIGURE 3:**
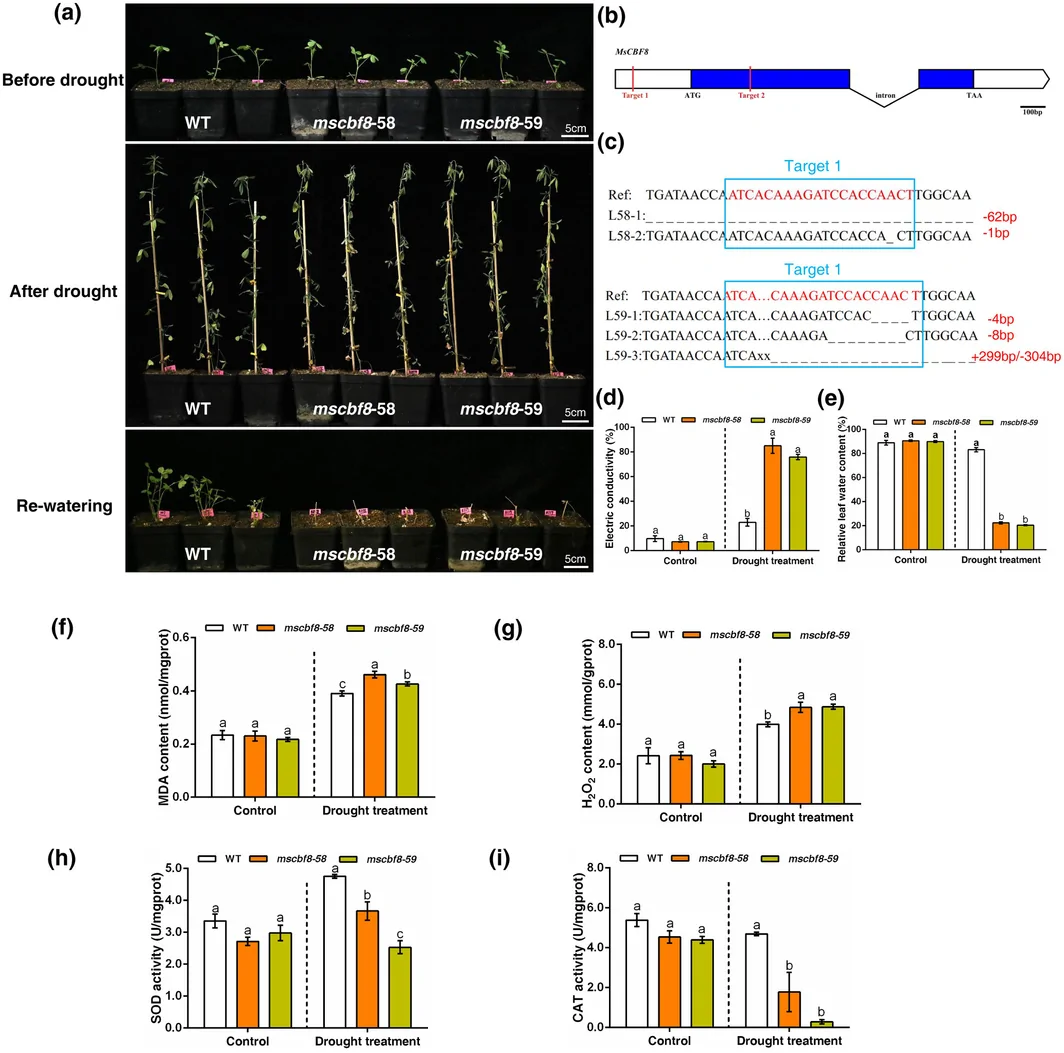
Alfalfa mutant *mscbf8* displayed elevated sensitivity to drought stress. (a) The growth performance of *mscbf8* and wild type (WT) plants before treatment, after natural drought treatment and re‐watering. The natural drought and rewatering methods are consistent as mentioned in Figure 2. (b) Schematic diagram of the gene structure model of *MsCBF8*. The target site was marked in red line. Coding sequences (CDS) are represented by blue boxes, introns by zigzag lines, with the 300‐bp upstream region preceding the ATG start codon and the 300‐bp downstream region following the TAA stop codon depicted as white boxes. (c) CRISPR‐Cas9‐induced mutations at the target Site 1 (ATCACAAAGATCCACCAACT) of *mscbf8‐58* and *mscbf8‐59* were verified through PCR amplification coupled with Sanger sequencing. Deletions are denoted by “_” symbols and insertions by “*x*” symbols, with the designations −1, −2 and −3 in the figure corresponding to distinct mutation types. (d–i) Electric conductivity, relative leaf water content, MDA, H_2_O_2_ content, SOD activity and CAT activity in *mscbf8* and WT after drought treatment. Error bars represent the mean ± standard error of the mean (SEM) from three biological replicates. Statistical significance between groups was determined by one‐way analysis of variance (ANOVA). Bars labelled with different lowercase letters indicate statistically significant differences at *p* < 0.05 based on Duncan's multiple range test.

To elucidate the role of *MsCBF8* in alfalfa drought tolerance, we generated mutants (*mscbf8‐58*, *mscbf8‐59* and *mscbf8‐9*) through CRISPR/Cas9‐mediated genome editing. The mutants were validated by PCR sequencing and single colony sequencing (Figures S5 and S6), revealing frameshift mutations featured by 1‐, 4‐, 6‐ or 8‐bp deletions and large‐fragment deletions/insertions (> 20 bp) at target sites (Figure 3c and Figures S5 and S6). qPCR analysis showed that the expression of *MsCBF8* was significantly reduced in the mutant lines, with transcript levels in *mscbf8‐58*, *mscbf8‐59* and *mscbf8‐9* decreased by 74%, 53% and 62%, respectively, compared with the wild type (Figure S6b). Following 18‐day growth in vermiculite, WT and mutant lines (*mscbf8‐58* and *mscbf8‐59*) were subjected to 30‐day natural drought. Post‐drought phenotypic analysis showed severe wilting and irreversible desiccation in mutants compared to WT (Figure 3a), with only 0% (*mscbf8‐58*) and 40% (*mscbf8‐59*) survival rates after 7‐day rehydration versus 80% in WT (Figure S7).

The electric conductivity of *mscbf8‐58* and *mscbf8‐59* leaves reached 85% and 76%, representing 62% and 53% increases, respectively, over WT (*p* < 0.05, Figure 3d), suggesting that the cell membrane of *mscbf8* mutants was damaged by drought stress. The relative water content decreased by 61%–63% in mutants compared to drought‐stressed WT (*p* < 0.05, Figure 3e), indicating that the leaf water holding capacity in *mscbf8* decreased. In addition, the measurement of oxidative damage indices showed that the MDA content in *mscbf8‐58* and *mscbf8‐59* increased significantly after drought stress (*p* < 0.05, Figure 3f), and the H_2_O_2_ levels rose by 21% compared to WT (*p* < 0.05, Figure 3g). The SOD activity in *mscbf8‐58* and *mscbf8‐59* was only 77% and 53% of WT, respectively (Figure 3h). Similarly, the CAT activity was also significantly reduced (*p* < 0.05, Figure 3i). These results demonstrate that *MsCBF8* mutation exacerbates drought‐induced cellular damage through impaired membrane integrity, compromised water retention, and attenuated antioxidant capacity.

### 2.4 MsCBF8 Consistently Enhances Drought Tolerance and Water‐Use Efficiency in Alfalfa Across Distinct Drought Stress Systems

**FIGURE 4:**
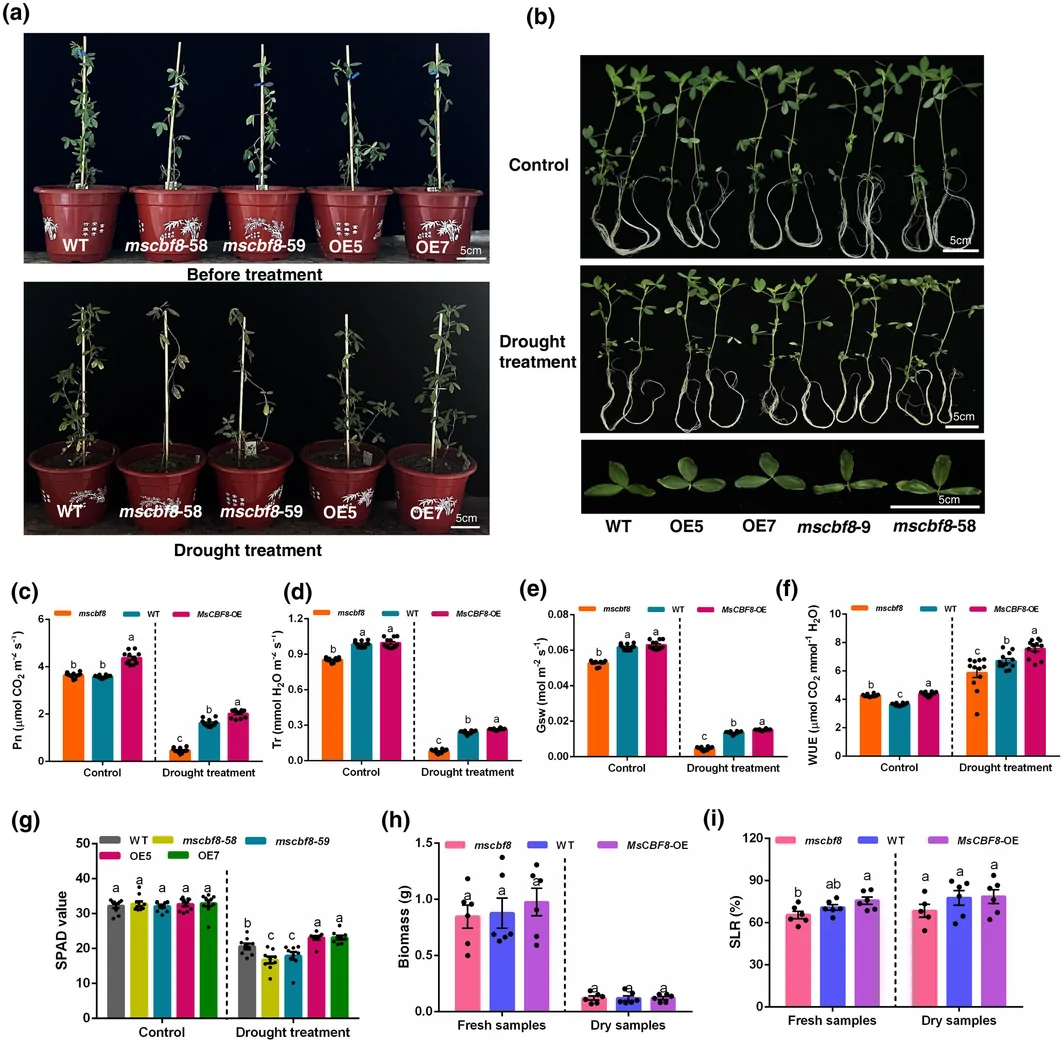
The impact of the *MsCBF8* gene on plant drought response and growth traits. (a) Growth phenotypes of WT, edited *mscbf8* mutants, and *MsCBF8*‐overexpressing (*MsCBF8*‐OE) plants after drought treatment 16 days. The experiment was conducted in a multi‐span greenhouse at the International High‐Tech Industrial Park of the Chinese Academy of Agricultural Sciences in Langfang, Hebei Province (39°35′47.03″ N, 116°35′16.24″ E), using topsoil sandy loam collected from the park, where light and temperature conditions more closely mimicked the natural environment. (b) Phenotypes of two‐week‐old hydroponically grown wild‐type, *MsCBF8*‐overexpressing and *mscbf8* CRISPR‐edited mutant plants after treatment with 100 mM mannitol for 2 days in a growth chamber. (c–f) Leaf net photosynthetic rate (Pn), transpiration rate (Tr), stomatal conductance (Gsw), and water use efficiency (WUE) under control and drought conditions. (g) SPAD values (indicating relative chlorophyll content) of each genotype after drought treatment. (h) Biomass comparison of each genotype under well‐watered conditions. (i) Stem‐to‐leaf ratio (SLR) comparison of each genotype under well‐watered conditions. Error bars represent the mean ± standard error of the mean (SEM) from at least six biological replicates. Statistical significance between groups was determined by one‐way analysis of variance (ANOVA). Bars labelled with different lowercase letters indicate statistically significant differences at *p* < 0.05 based on Duncan's multiple range test.

To assess the function of MsCBF8 under more environmentally relevant conditions, we conducted soil‐based drought trials in a multi‐span greenhouse that simulated natural fluctuations in light and temperature (Figure 4a). In parallel, for precise verification under controlled conditions, we subjected the same set of materials to mannitol‐induced osmotic stress in growth chambers (Figure 4b). In both systems, *MsCBF8*‐overexpressing (OE) lines exhibited the highest drought tolerance and growth performance, followed by the wild‐type (WT), whereas *mscbf8* mutants displayed pronounced wilting and damage (Figure 4a,b).

In‐depth analysis of post‐drought leaf photosynthetic physiology revealed a consistent regulatory pattern. The net photosynthetic rate (Pn), stomatal conductance (Gsw) and transpiration rate (Tr) were all significantly higher in OE lines compared to WT and mutants (Figure 4c–e). Crucially, the water‐use efficiency (WUE) of OE lines was 1.3‐fold and 1.1‐fold that of the mutant and WT, respectively (Figure 4f), indicating that MsCBF8 enhances carbon assimilation efficiency while reducing water loss.

Furthermore, although chlorophyll content (SPAD value) did not differ among genotypes prior to drought stress, OE lines showed the greatest chlorophyll retention after stress, followed by WT, while mutants suffered a significant decline (Figure 4g). It is noteworthy that under well‐watered conditions, no significant differences were observed in biomass accumulation or shoot‐to‐leaf ratio (SLR) among the lines in dry samples (Figure 4h,i), demonstrating that *MsCBF8* overexpression does not compromise basal growth but specifically optimizes physiological adaptation under drought stress.

### 2.5 Complementation in Arabidopsis Confirms the Conserved Drought‐Tolerance Function of MsCBF8

**FIGURE 5:**
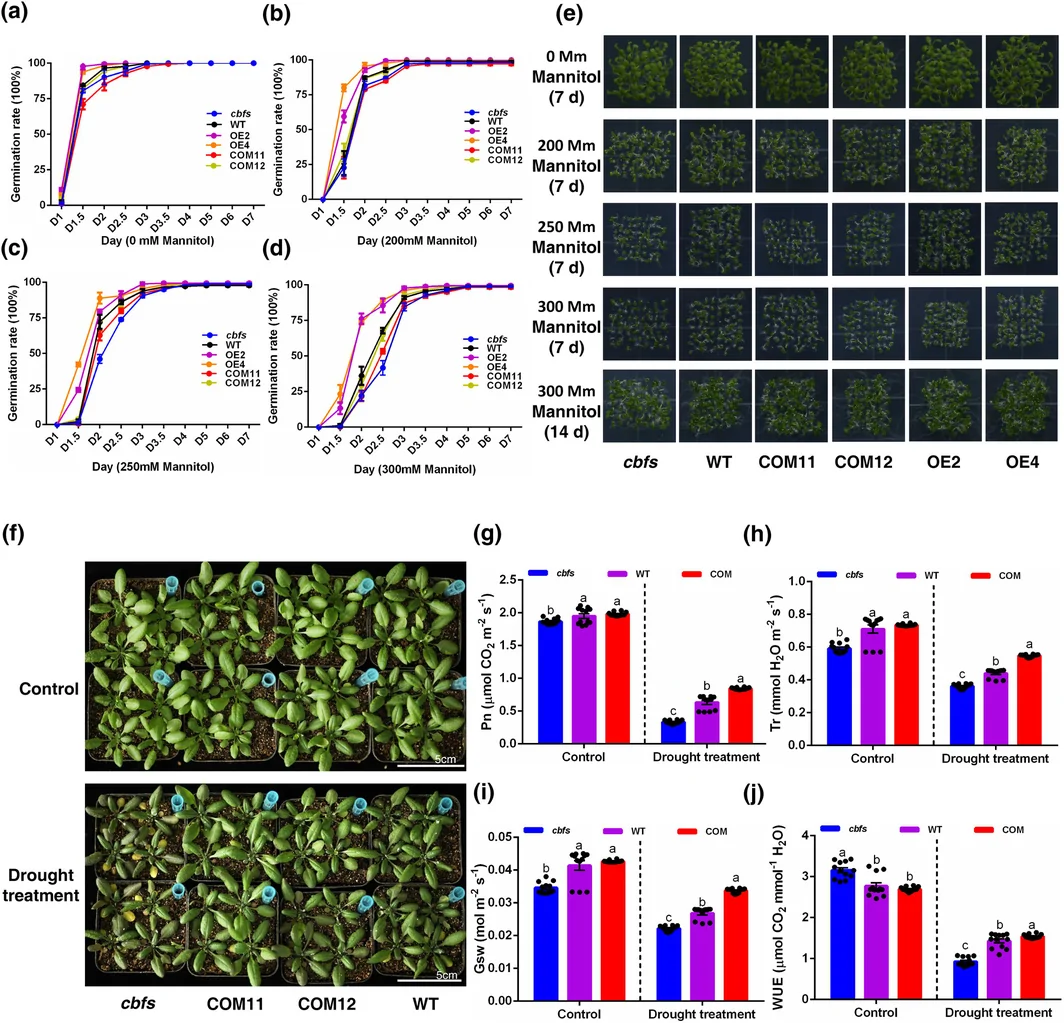
Functional analysis of drought tolerance in Arabidopsis following *MsCBF8* complementation. (a–d) Germination phenotypes of Arabidopsis seeds from the *cbfs* mutant, WT, *MsCBF8*‐overexpressing lines (OE2 and OE4), and mutant‐complemented lines (COM11 and COM12) on media containing different concentrations of mannitol (0, 200, 250 and 300 mM). (e) Phenotypic comparison of the aforementioned lines grown on media with different mannitol concentrations at 7 and 14 days after sowing. (f) Phenotypes of two‐week‐old seedlings from different genotypes (mutant, complementation lines and wild‐type WT) after simulated drought treatment with mannitol (50 mM for 5 days followed by 100 mM for 2 days). (g‐j) Net photosynthetic rate (Pn), transpiration rate (Tr), stomatal conductance (Gsw), and water‐use efficiency (WUE) in leaves of the different Arabidopsis lines under drought conditions. Error bars represent the mean ± standard error of the mean (SEM) from at least three biological replicates. Statistical significance between groups was determined by one‐way analysis of variance (ANOVA). Bars labelled with different lowercase letters indicate statistically significant differences at *p* < 0.05 based on Duncan's multiple range test.

To obtain more definitive evidence, we performed functional complementation assays in Arabidopsis. First, we analysed the response of seed germination to osmotic stress on media containing different concentrations of mannitol (0, 200, 250 and 300 mM). Materials tested included the *cbfs* mutant, wild‐type (WT), two homozygous *MsCBF8*‐overexpressing lines (OE2 and OE4), and two homozygous complementation lines (COM11 and COM12) (Figure 5a–d). Germination kinetics revealed that genotypic differences were primarily evident during the early phase (1.5–3 days), after which germination rates plateaued (Figure 5e). Under normal (0 mM) and mild stress (200 mM) conditions, the germination rates of complementation lines showed no significant advantage over the *cbfs* mutant (Figure 5a,b). However, as stress severity increased, the complementation effect became pronounced: after 2 days on 250 mM mannitol, the germination rate of *cbfs* was only 46%, whereas WT, COM11 and COM12 reached 72%, 63% and 72%, respectively. The overexpression lines performed best, achieving 79% and 89% (Figure 5c). Under severe osmotic stress (300 mM), the complementation lines consistently outperformed the *cbfs* mutant, with the overexpression lines again showing the highest germination rates (Figure 5d). These results collectively demonstrate that *MsCBF8* significantly enhances seed germination under osmotic stress in Arabidopsis, indicating a conserved function.

Seedlings were subsequently subjected to a sequential drought‐simulation treatment (50 mM mannitol for 5 days followed by 100 mM for 2 days) (Figure 5f). The *cbfs* mutant displayed severe leaf yellowing, purpling and thinning, while complementation lines (COM11 and COM12) and WT exhibited markedly less damage (Figure 5f). This phenotypic difference correlated with chlorophyll content (Figure S8a–c). Photosynthetic parameters measured after stress showed that complementation lines had significantly higher net photosynthetic rate (Pn), transpiration rate (Tr), stomatal conductance (Gsw) and water‐use efficiency (WUE) than WT and were substantially superior to the *cbfs* mutant (Figure 5g–j). Oxidative stress indicators further supported the complementation effect: the *cbfs* mutant accumulated the highest levels of MDA and H_2_O_2_ (Figure S8d,e) and exhibited the lowest activities of SOD and CAT (Figure S8f,g). Complementation with *MsCBF8* effectively mitigated these physiological imbalances. In summary, complementation with *MsCBF8* in Arabidopsis not only restored the drought tolerance of the *cbfs* mutant but even surpassed the wild‐type level, comprehensively demonstrating the essential and conserved role of MsCBF8 in enhancing plant drought resistance from seed germination to seedling growth.

### 2.6 MsCBF8 Coordinates Dynamic Stomatal Aperture Regulation and Drives the Transcriptional Cascade of Drought‐Responsive Genes

**FIGURE 6:**
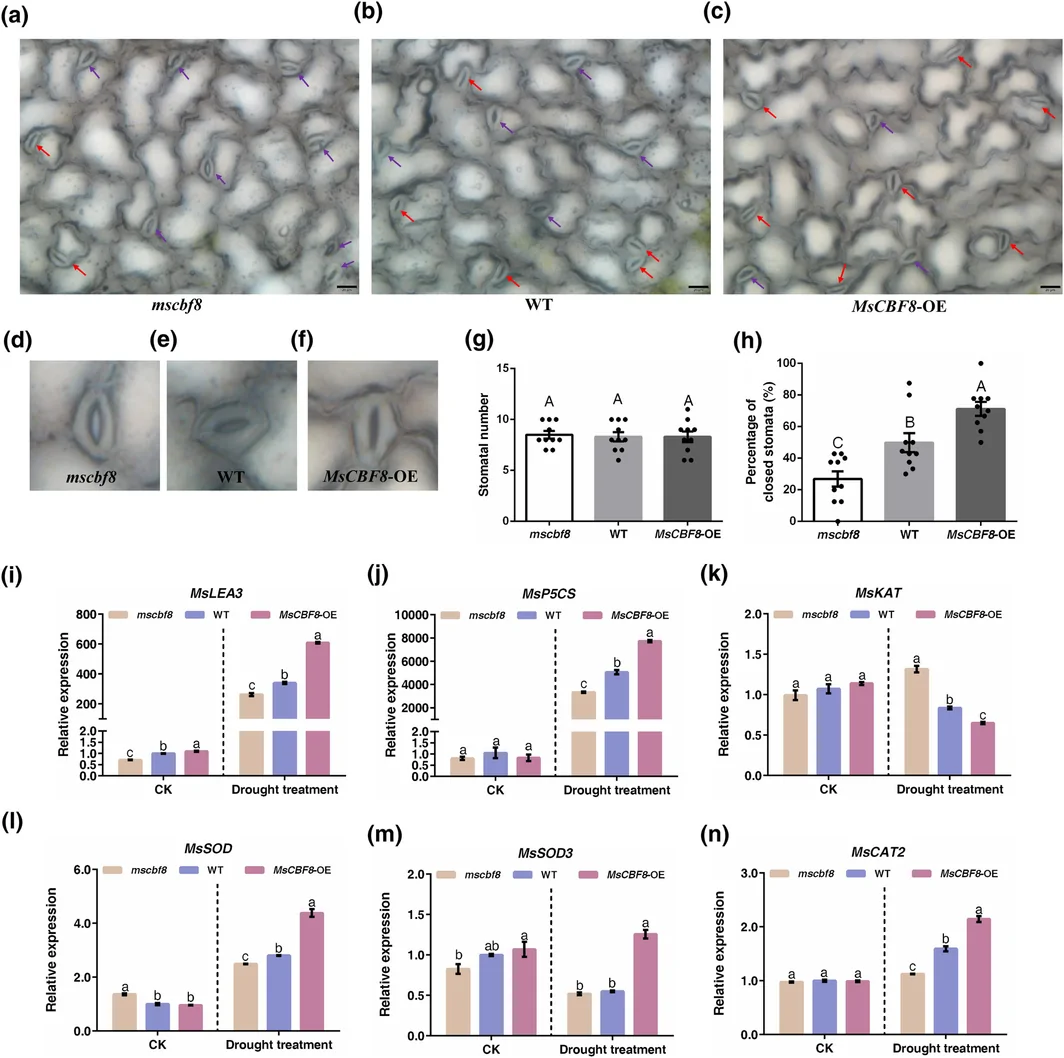
Dynamic regulation of stomatal aperture and expression profiles of stress‐responsive genes in leaves of *mscbf8* mutants, WT and *MsCBF8*‐overexpressing plants under drought stress. (a) and (d) Dynamic regulation of stomatal aperture in *mscbf8* under 150 mM mannitol treatment. (b) and (e) Dynamic regulation of stomatal aperture in WT under 150 mM mannitol treatment. (c) and (f) Dynamic regulation of stomatal aperture in *MsCBF8*‐OE under 150 mM mannitol treatment. Bar = 20 μm. (g) Stomatal number statistics. (h) Percentage of closed stomata in *mscbf8*, WT and *MsCBF8*‐OE leaves under 150 mM mannitol treatment. Red arrows represent closed stomata, and purple arrows represent opening stomata. (i–n) The relative expression level of *late embryogenesis abundant group 3* (*MsLEA3*), *pyrroline‐5‐carboxylate synthase* (*MsP5CS*), *potassium channel KAT* (*MsKAT*), *superoxide dismutase* (*MsSOD* and *MsSOD3*), *catalase 2* (*MsCAT2*) in edited mutants, wild‐type and overexpression lines after drought treatment. Error bars represent the mean ± standard error of the mean (SEM) from three biological replicates. Statistical significance between groups was determined by one‐way analysis of variance (ANOVA). Bars labelled with different lowercase letters indicate statistically significant differences at *p* < 0.05 based on Duncan's multiple range test.

Physiological characterization revealed differential water retention capacities among overexpression, WT and mutant plants under drought stress (Figures 2d and 3e). To investigate stomatal regulation mechanisms, we quantified stomatal aperture dynamics under 150 mM mannitol‐induced drought simulation. While total stomatal density remained comparable across all genotypes (Figure 6g), critical differences emerged in stomatal closure patterns: 71% of stomata were closed in *MsCBF8‐*OE under drought stress, while closure rates in WT and *mscbf8* were only 50% and 27%, respectively (Figure 6). This drought‐induced stomatal closure advantage in *MsCBF8‐*OE plants over WT and mutant *mscbf8* demonstrates that *MsCBF8* enhances drought tolerance through coordinated regulation of stomatal aperture and water retention.

Our physiological assessments established the central role of MsCBF8 in drought adaptation through osmotic adjustment, ROS detoxification and stomatal regulation, prompting systematic evaluation of associated molecular markers. Under 150 mM mannitol‐induced drought (24 h), qRT‐PCR analyses revealed that the stress‐responsive gene *MsLEA3* and proline biosynthesis gene *MsP5CS* exhibited a maximal induction of expression levels in *MsCBF8*‐OE lines (*p* < 0.05), followed by WT and a lowest induction in mutants *mscbf8* (Figure 6i,j). Concurrently, the stomatal closure‐associated potassium channel gene *MsKAT* displayed an opposite expression pattern, with minimal transcripts in OE lines (0.8‐fold of WT) and maximal accumulation in mutants (1.6‐fold of WT), aligning with observed stomatal aperture phenotypes (Figure 6k). Furthermore, antioxidant genes *MsSOD*, *MsSOD3* and *MsCAT2* demonstrate stress‐inducible expression hierarchies paralleling enzymatic activity enhancements (OE > WT > mutants), exhibiting 1.3–2.4‐fold upregulation in *MsCBF8*‐OE lines (Figure 6l–n). These results indicate that MsCBF8 orchestrates drought tolerance by activating a transcriptional cascade of stress‐responsive genes.

### 2.7 *MsGolS2* Is a Direct Downstream Target of MsCBF8 and Contributes to Drought Tolerance in Alfalfa

**FIGURE 7:**
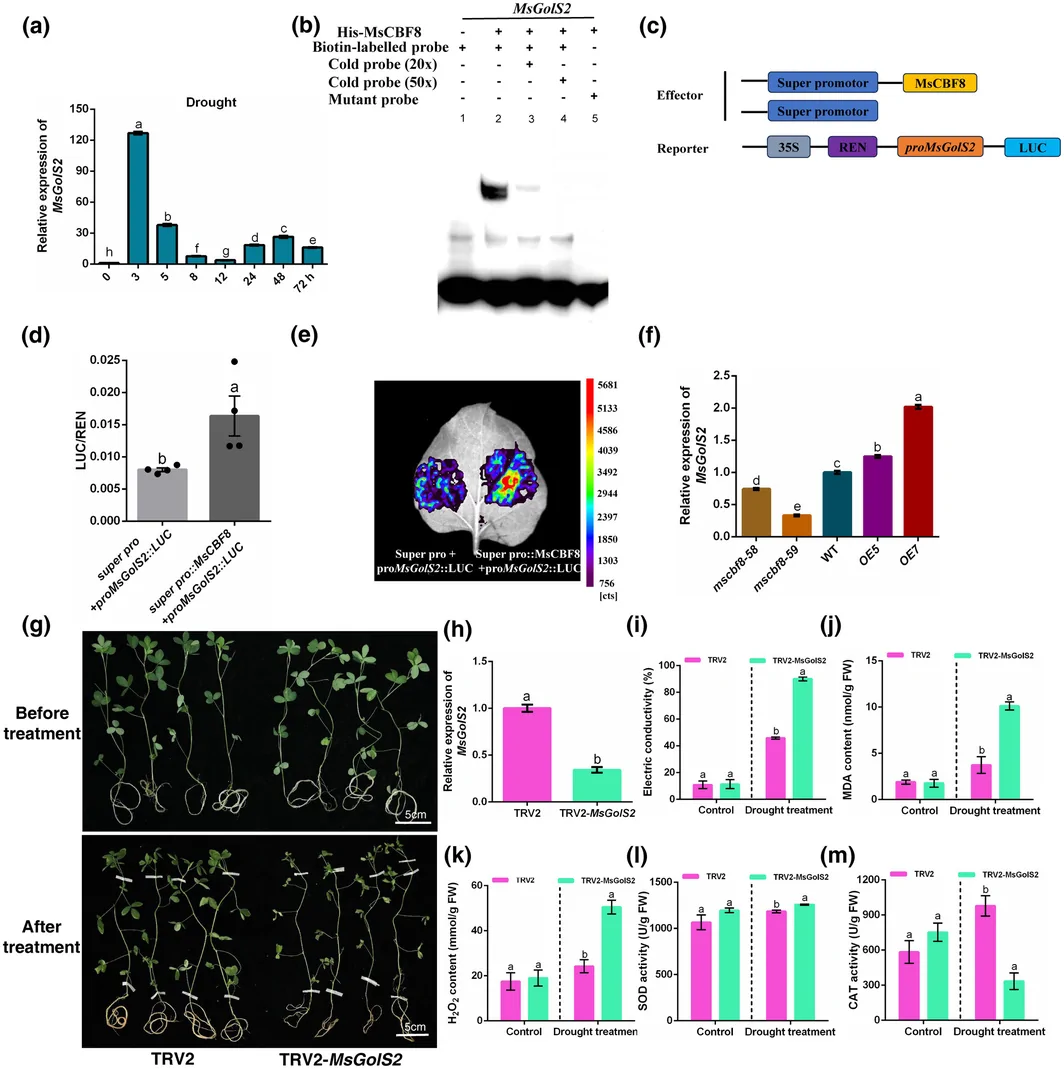
*MsGolS2* acts as a downstream target gene of MsCBF8 to positively regulate drought tolerance in alfalfa. (a) Time‐course expression analysis of *MsGolS2* under drought stress simulated by 15% PEG6000. (b) Electrophoretic Mobility Shift Assay (EMSA) was performed to detect the binding characteristics between purified His‐MsCBF8 fusion protein and biotin‐labelled *MsGolS2* promoter probe, where “+” and “−” indicate the presence and absence of corresponding components in the reaction system, respectively. (c) Schematic diagram of the effector and reporter constructs used for the dual‐luciferase assay. (d) Dual‐luciferase reporter assay showing MsCBF8 regulated and enhanced the transcription of *MsGolS2*. (e) LUC fluorescence imaging in tobacco leaves after transient co‐expression of the effector and reporter constructs. (f) Endogenous expression levels of *MsGolS2* in *mscbf8* mutants, WT and *MsCBF8*‐OE lines. (g) Growth phenotypes of *MsGolS2*‐knockdown lines (TRV2‐*MsGolS2*) compared with control plants carrying the empty TRV2 vector (CK) before and after simulated drought treatment (100 mM mannitol for 2 days). Treatment was applied to 4‐week‐old plants. (h) Expression analysis confirming the knockdown efficiency of *MsGolS2* in the silenced lines using TRV2 plants as control. (i–m) Physiological changes in electric conductivity, MDA, H_2_O_2_ content, and SOD, CAT activities in leaves of *MsGolS2*‐knockdown lines after drought treatment. Error bars represent the mean ± standard error of the mean (SEM) from at least three biological replicates. Statistical significance between groups was determined by one‐way analysis of variance (ANOVA). Bars labelled with different lowercase letters indicate statistically significant differences at *p* < 0.05 based on Duncan's multiple range test.

To elucidate the downstream regulatory network of MsCBF8, we investigated *MsGolS2*, which encodes a drought‐responsive galactinol synthase implicated in raffinose biosynthesis. Time‐course expression analysis showed that *MsGolS2* was rapidly and strongly induced under drought stress simulated by 15% PEG6000 (Figure 7a). Bioinformatic examination of its promoter region identified a DRE (GCCGAC) *cis*‐element, suggesting that *MsGolS2* could be a direct target of the DREB‐type transcription factor MsCBF8.

We first tested this interaction in vitro using an electrophoretic mobility shift assay (EMSA). Purified His‐MsCBF8 protein specifically bound to a biotin‐labelled probe containing the GCCGAC motif from the *MsGolS2* promoter. This binding was effectively competed by an unlabeled cold probe in a dose‐dependent manner, whereas a mutated probe failed to bind, confirming the sequence‐specific nature of the interaction (Figure 7b). We then assessed the transcriptional activation activity in planta using a dual‐luciferase reporter system in tobacco (*Nicotiana benthamiana*) leaves. Co‐expression of the MsCBF8 effector with the *MsGolS2* promoter‐driven reporter (Figure 7c) resulted in a significant increase in the LUC/REN ratio compared with controls (Figure 7c,d), which was visually corroborated by stronger LUC fluorescence (Figure 7e). These results demonstrate that MsCBF8 directly and specifically binds to the promoter and activates the expression of *MsGolS2*. Furthermore, endogenous *MsGolS2* transcript levels were significantly higher in *MsCBF8*‐OE lines and lower in *mscbf8* mutants relative to the wild‐type (WT) (Figure 7f), consistent with the notion of MsCBF8 acting as an upstream regulator of *MsGolS2*.

Given that the function of *GolS*‐related genes in alfalfa remains unexplored, we next determined the functional importance of *MsGolS2* in drought tolerance by silencing *MsGolS2* via virus‐induced gene silencing (VIGS). The expression level of *MsGolS2* in silenced lines was reduced by 66% compared with empty‐vector (TRV2) control plants (CK) (Figure 7h). After treatment with 100 mM mannitol for 2 days, the silenced lines exhibited more severe leaf wilting and shrinkage than the control (Figure 7g). Physiological analysis of stressed leaves revealed that knockdown of *MsGolS2* led to increased membrane damage, as indicated by elevated electrolyte leakage (Figure 7i) and MDA content (Figure 7j). Moreover, silenced plants accumulated more H_2_O_2_ (Figure 7k) and displayed reduced activities of SOD and CAT (Figure 7l,m), indicating aggravated oxidative damage.

In summary, these results prove *MsGolS2* as a direct transcriptional target of MsCBF8 in alfalfa and demonstrate that *MsGolS2* functions as a positive regulator in the MsCBF8‐mediated drought‐tolerance pathway, partly through alleviating oxidative and membrane damage.

## 3 Discussion

### 3.1 MsCBF8 Is a Key Drought‐Tolerance Regulator Without Growth Penalty in Alfalfa

The CBF/DREB1 transcription factor family has been widely recognized as pivotal regulators in plant abiotic stress responses. While CBF/DREB1 is traditionally associated with cold acclimation (Liu et al. 1998; Kidokoro et al. 2022), their functional mechanisms in drought adaptation have not been fully elucidated. In this study, *k*‐mer‐based GWAS was used to identify a novel drought‐responsive locus encoding a CBF homologue in alfalfa. Notably, this locus co‐localizes with a known fall‐dormancy QTL (Zhang, Kang, et al. 2023). Based on multiple sequence alignment and phylogenetic analysis, we designated this gene *MsCBF8*.

However, functional validation of candidate genes in autotetraploid alfalfa imposes a notable technical hurdle due to its complex, highly repetitive genome and the presence of multiple homeologous copies. Genetic engineering in alfalfa has historically been hindered by low editing efficiency, with early CRISPR systems achieving only modest rates (3%–8%) in alfalfa (Shi, Dong, et al. 2024). Recent breakthroughs in vector design, particularly using optimized tRNA‐sgRNA scaffolds, have elevated editing efficiency above 35% (Ye et al. 2022; Zhao et al. 2024). Leveraging this optimized CRISPR 2.0 toolkit (Zhao et al. 2024), we successfully generated *MsCBF8* mutants. CRISPR/Cas9‐mediated editing yielded a mosaic of mutations across the four homeologs with varying efficiency (17%–77% per copy), which nonetheless sufficed to create loss‐of‐function lines with significantly reduced *MsCBF8* expression (53%–74% knockdown) (Figure 3 and Figures S5 and S6). The pronounced drought hypersensitivity observed in these partially edited mutants underscores the critical role of *MsCBF8* and demonstrates that haploinsufficiency can be exploited for functional studies in polyploids, where obtaining complete, homozygous knockouts remains exceptionally challenging. Conversely, overexpression of *MsCBF8* consistently enhanced drought tolerance across independent events and diverse environments (Figures 2 and 4). This robust, dosage‐dependent phenotypic response, coupled with successful cross‐species complementation in Arabidopsis (Figure 5), firmly establishes MsCBF8 as a validated drought‐tolerance gene with conserved function across plants.

Critically, and in contrast to some stress tolerance mechanisms that incur growth penalties (Chen et al. 2010; Li et al. 2018; Li et al. 2022), *MsCBF8* overexpression enhanced drought tolerance without compromising biomass accumulation or shoot‐to‐leaf ratio under well‐watered conditions (Figure 4h,i). Moreover, we found that MsCBF8 also enhances freezing tolerance in alfalfa (Figure S9). The combination of stress tolerance without growth penalty positions MsCBF8 as a prime breeding target, and our work—overcoming functional genomics hurdles in alfalfa—establishes it as a key regulator for sustaining yield under drought.

### 3.2 MsCBF8 Orchestrates an Integrated Defence Module Conferring Drought Tolerance in Alfalfa

Drought stress imposes dual assaults on plant cells through membrane destabilization and oxidative burst, necessitating coordinated protective mechanisms (Ferreira et al. 2021; Dutta et al. 2024). In line with the reported functions of DREB1 orthologs in species such as banana (Xu et al. 2022), oil palm ( *Elaeis guineensis* ) (Azzeme et al. 2017) and rice ( *Oryza sativa* ) (Wang et al. 2022), our study demonstrates that *MsCBF8*‐overexpressing (OE) lines maintain membrane and redox homeostasis under drought (Figure 2). In contrast, *mscbf8* mutants exhibit aggravated oxidative damage and compromised antioxidant capacity (Figure 3). Similarly, alfalfa studies demonstrate that stress‐responsive genes like *MsCYP71* and *MsMYBH* regulate water balance, maintain photosynthesis and boost ROS scavenging (Liu, Shi, et al. 2023; Shi, Liu, et al. 2024). At the transcriptional level, MsCBF8 simultaneously upregulates the osmo‐protective gene *MsLEA3* and antioxidant genes to neutralize ROS (Figure 6), thereby synergistically stabilizing cellular membranes and scavenging reactive oxygen species to achieve comprehensive cellular protection.

Stomatal dynamics, serving as a critical nexus between water conservation and photosynthetic efficiency, are finely tuned by environmental stressors including water deficit and ROS accumulation. Under drought conditions, plants reduce water transpiration by regulating stomatal closure to improve drought tolerance (Schroeder et al. 2001; Qi et al. 2018; He, Wang, et al. 2025). Recent studies have demonstrated that overexpression of *MtNAC33* in alfalfa enhances drought tolerance by orchestrating stomatal closure through transcriptional reprogramming of key stomatal movement‐associated genes (e.g., *KAT*), thereby reducing transpiration water loss while maintaining photosynthetic efficiency (Yang et al. 2025). Notably, our physiological analyses revealed that MsCBF8 precisely enhances stomatal closure under osmotic stress (71% in OE vs. 50% in WT), thus improving water retention (RWC increased by 67%–71% over WT). These findings parallel observations in Arabidopsis drought‐sensitive mutants: *gad12* (Mekonnen et al. 2016), *cop1‐4* and *lip1* mutants (Moazzam‐Jazi et al. 2018) display defective stomatal closure. However, during extended drought, *MsCBF8*‐OE plants maintain higher rates of net photosynthesis, transpiration and stomatal conductance, ultimately achieving superior water‐use efficiency. This indicates a dynamically coordinated physiology in which MsCBF8 both heightens stomatal sensitivity to conserve water and enhances mesophyll photosynthetic capacity, thus optimizing the carbon‐water balance under drought.

### 3.3 The MsCBF8‐ *MsGolS2* Module: A Novel Regulatory Module in Alfalfa Drought Tolerance

Soluble sugars, particularly raffinose family oligosaccharides (RFOs), play a key role in osmotic adjustment under drought stress (Guo et al. 2021; Liu, Li, et al. 2023; Cui et al. 2024). Galactinol synthase (GolS) catalyses a key step in the raffinose biosynthetic pathway. Three stress‐responsive *GolS* genes, namely *AtGolS1*, *AtGolS2* and *AtGolS3*, have been identified in Arabidopsis. It has been demonstrated that overexpression of *AtGolS2* and *ZmGolS2* in Arabidopsis enhances plant drought tolerance (Taji et al. 2002; Gu et al. 2016). Although DREB‐type transcription factors are known to regulate *GolS* expression (Gu et al. 2016)—for example, DREB2A activates *GOLS2* in maize, whether and how CBF/DREB1 family members modulate drought tolerance via this pathway, especially in perennial legume forages, remains unclear.

Our study fills this knowledge gap by identifying *MsGolS2* as a downstream target of a CBF/DREB1 member, MsCBF8, in alfalfa. We found that *MsGolS2*, which is strongly induced by drought, contains a canonical GCCGAC *cis*‐element in its promoter. EMSA and dual‐luciferase reporter assays confirmed that MsCBF8 directly binds to this element and activates *MsGolS2* transcription. Consistently, *MsGolS2* expression was upregulated in *MsCBF8*‐overexpression lines and down‐regulated in *mscbf8* mutants, establishing a direct transcriptional regulatory relationship (Figure 7a–f).

Although the importance of GolS in plant stress adaptation has been noticed (Wang et al. 2021; Zhang, He, et al. 2023; Chen et al. 2025), the function of *GolS* family genes has not been previously reported in alfalfa. To verify whether *MsGolS2* is involved in drought tolerance, we generated *MsGolS2*‐knockdown lines via virus‐induced gene silencing (VIGS). Compared with controls, silenced plants showed significantly reduced drought tolerance, accompanied by aggravated oxidative damage (elevated H_2_O_2_ and MDA contents) and diminished antioxidant capacity (decreased SOD and CAT activities) (Figure 7g–m). These results provide the first functional evidence that *MsGolS2* is essential for drought tolerance in alfalfa and that its activity mitigates oxidative and membrane damage.

**FIGURE 8:**
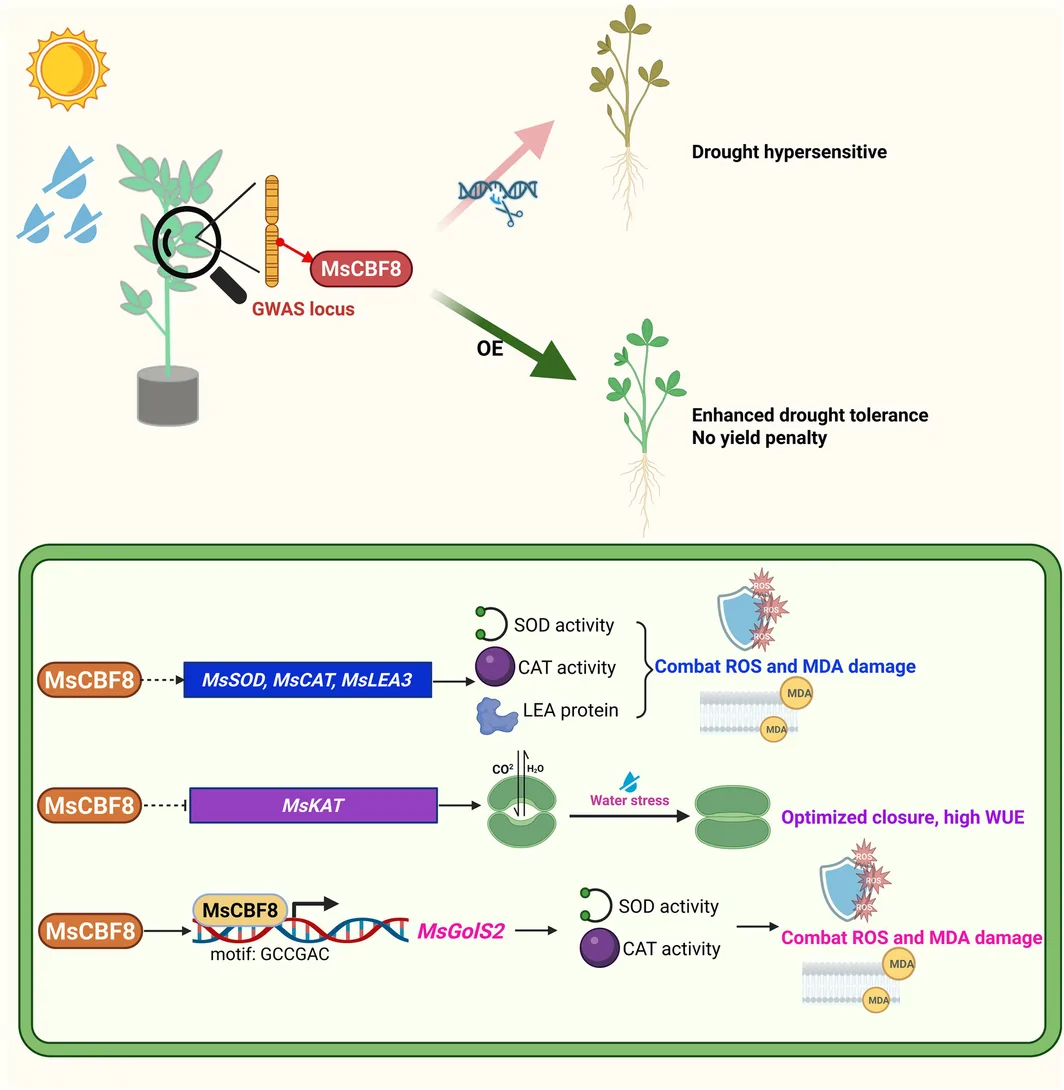
A working model illustrating the multilayer defence network orchestrated by MsCBF8 to confer drought tolerance in alfalfa. The schematic diagram was created with BioRender.com (under an academic licence).

Collectively, this study establishes MsCBF8 as a pivotal and agronomically favourable key regulator of drought tolerance in autotetraploid alfalfa. By leveraging an optimized CRISPR toolkit to overcome functional genomics challenges in this polyploid crop, we demonstrate that MsCBF8 enhances drought resilience without incurring growth penalties. Mechanistically, MsCBF8 orchestrates a multi‐layered defence network: it coordinates antioxidant defence and membrane protection, dynamically optimizes stomatal conductance to improve water‐use efficiency, and directly activates the raffinose pathway gene *MsGolS2* by binding to its promoter—thereby defining a novel MsCBF8‐*MsGolS2* regulatory module (Figure 8). Although the current research results are based on controlled experimental conditions, future multi‐environment field trials will further verify the stability of MsCBF8‐mediated drought tolerance and its effect on yield and quality, and the use of drought‐inducible or tissue‐specific promoters to carry out refined functional exploration will promote its practical application in breeding drought‐tolerant crops. These findings deepen the molecular understanding of drought adaptation in forage legumes and provide a valuable genetic module for breeding high‐yield, drought‐tolerant alfalfa varieties.

## 4 Experimental Procedures

### 4.1 Plant Materials and Growth Conditions

Two plant species were used in this study: alfalfa ( *Medicago sativa* L. ‘Zhongmu No. 1’) and Arabidopsis ( *Arabidopsis thaliana* ecotype Columbia‐0, Col‐0). Plants were cultivated under three distinct regimes:

1. Soil culture in controlled growth chambers: Conducted in growth chambers at the Institute of Animal Sciences, Chinese Academy of Agricultural Sciences (Beijing). Plants were grown in an artificial soil mixture (vermiculite: nutrient soil = 2:1). Chamber conditions were set to a 24°C/22°C (day/night) temperature regime, a 16‐h photoperiod with a photosynthetic photon flux density of 65 mmol m^−2^ s^−1^, and 60%–70% relative humidity.
2. Soil culture in a multi‐span greenhouse: Conducted in a multi‐span greenhouse at the International High‐Tech Industrial Park of the Chinese Academy of Agricultural Sciences (Langfang, Hebei; 39°35′ 47.03″ N, 116° 35′ 16.24″ E). Plants were grown in sandy loam soil collected from the site. The greenhouse provided natural fluctuations in light and temperature. Experiments were performed in November 2025.
3. Hydroponic culture in growth chambers: Conducted in the aforementioned controlled growth chambers. Plants were grown using half‐strength Hoagland nutrient solution.

### 4.2 Genome‐Wide Association Analysis

Drought tolerance evaluation was performed on 171 alfalfa cultivars under controlled conditions as previously described (Jiang et al. 2025). We quantified stem‐to‐leaf ratio (SLR) in both well‐watered and drought‐stressed treatments. To systematically assess drought responsiveness, the Drought Response Value (DRV) was calculated using the formula:

DRV = (SLR‐control condition − SLR‐drought condition)/SLR‐control condition.

Subsequent genome‐wide association analysis (GWAS) was conducted using DRV as the phenotypic input. GWAS analyses based on SNPs and indels are critically dependent on the assembly quality of the reference genome. Furthermore, due to the complex autopolyploid genomic characteristics in alfalfa, these methods exhibit substantial limitations in alignment processes, failing to effectively detect variations among homologous chromosomes. To overcome these limitations, we implemented a *k*‐mer‐based association analysis using KMERIA (https://github.com/Sh1ne111/KMERIA). The individual *k*‐mer (31‐bp) profiles were initially derived from the raw sequencing data and then consolidated into a population‐level *k*‐mer count matrix. This procedure generated 489 files, each containing 10 million *k*‐mers. For each file, *k*‐mers with a missing rate > 20% or an abundance > 1000 were excluded, and the filtered matrix was converted into BIMBAM format using the ‘m2b’ command. A random set of 20 000 *k*‐mers per file was selected for principal component analysis (PCA) performed with Plink (Purcell et al. 2007) and kinship matrix estimation using GEMMA (Zhou and Stephens 2012). Genome‐wide association analysis was conducted under a linear mixed model. *k*‐mers exceeding the significance threshold of *p* < 1 × 10^−4^ were aligned to the reference genome via blastn for subsequent visualization in a Manhattan plot. In the *k*‐mer‐based association analysis, *k*‐mers meeting the threshold of −log_10_(*p*) ≥ 7 were classified as statistically significant markers (Table S2).

### 4.3 Evolutionary Relationship Analysis and Protein Structural Analysis of MsCBF8

A phylogenetic tree was constructed using the MEGA 7.0 software. Signal peptide identification was achieved using SignalP 5.0 (https://services.healthtech.dtu.dk/services/SignalP‐5.0/). Secondary structure analysis was implemented in SOPMA (https://npsa.lyon.inserm.fr/cgi‐bin/npsa_automat.pl?page=/NPSA/npsa_sopma.html). Conserved domain analysis of the protein was performed on NCBI.

### 4.4 Total RNA Isolation and Reverse Transcription

Total RNA was isolated from alfalfa using the Eastep Total RNA Extraction Kit (Promega, USA). First‐strand cDNA synthesis was performed with 1 μg of total RNA using the HiScript III All‐in‐One RT SuperMix kit (Vazyme, China) following the manufacturer's protocols. Quantitative real‐time PCR analysis was conducted on a CFX96 Touch Real‐Time PCR System (Bio‐Rad) with Taq Pro Universal SYBR qPCR Master Mix (Vazyme, China).

### 4.5 Autonomous Transcriptional Activation Assay

The coding sequence of *MsCBF8* was fused to the GAL4 DNA‐binding domain (BD) of the pGBKT7 bait vector (Clontech) through *EcoR* I restriction sites. Verified recombinant plasmids were transformed into Y2HGold yeast competent cells (Coolaber) using the lithium acetate method. For yeast two‐hybrid analysis, the growth state of co‐transformants with BD‐MsCBF8 and pGADT7 (AD)‐fusion constructs was examined on SD/−Trp‐Leu (DDO) and SD/−Trp‐Leu‐His‐Ade (QDO) media. In addition, three control groups were included: (1) Positive control: BD‐53 + AD‐T; (2) Negative control: BD‐lam + AD‐T; (3) Double negative control: BD + AD. All plates were incubated at 30°C for 5–7 days with daily documentation of colony growth.

### 4.6 Generation of Alfalfa *MsCBF8* Overexpression Lines and Arabidopsis Mutant Complementation Materials

The *MsCBF8* overexpression construct was generated by inserting the full‐length coding sequence into the Super1300 vector (Li et al. 2001) using *Sma* I restriction sites. Specifically, the Super::*MsCBF8*‐OE5 and Super::*MsCBF8*‐OE7 lines were generated by cloning the coding sequence of *MsCBF8* into the *Sma* I site of the Super1300 vector. Verified recombinant plasmids were transformed into the *Agrobacterium tumefaciens* strain EHA105 (for alfalfa) or GV3101 (for Arabidopsis). Alfalfa genetic transformation was conducted following Fu's protocol (Fu et al. 2015). Transgenic Arabidopsis plants were generated via the floral dip transformation method (Clough and Bent 1998). Primary screening was conducted by PCR amplification using vector‐specific primers (MsCBF8‐1300F and 1300R). Transcript levels were quantified via qRT‐PCR with gene‐specific primers (MsCBF8‐RT‐F/R) with *MsACTIN* or *AtACTIN* as the endogenous control. Two overexpression lines (*MsCBF8*‐OE5 and *MsCBF8*‐OE7) were vegetatively propagated for phenotypic characterization.

### 4.7 Generation of CRISPR‐Cas9‐Edited Mutant *mscbf8*

CRISPR guide RNAs (sgRNAs) targeting *MsCBF8* were designed using the AlfalfaGenome sgRNA Design Platform (http://alfalfagedb.liu‐lab.com/target/sgRNA_target/). The sgRNA expression cassettes were cloned into the CRISPR 2.0 vector (Zhao et al. 2024). Verified constructs were introduced into the *Agrobacterium tumefaciens* strain EHA105. Similarly, alfalfa genetic transformation was conducted following Fu's protocol (Fu et al. 2015). Putative mutants were verified through a suite of confirmation protocols: (1) PCR amplification with Cas9‐specific primers (Table S1) followed by Sanger sequencing (Tsingke Biotechnology), (2) TA cloning of target region amplicons into pCE2 TA/Blunt‐Zero vector (Vazyme) for indel analysis using the SnapGene software, and (3) quantification of *MsCBF8* expression in promoter‐edited lines. Three independent mutant lines (*mscbf9*, *mscbf58* and *mscbf59*) were propagated for phenotypic characterization.

### 4.8 Expression Analysis Under Stress

Uniformly sized and plump alfalfa seeds were surface‐sterilized and germinated on moist filter paper in Petri dishes. After 7 days of growth under controlled conditions, seedlings with consistent developmental stages were transferred to 1/2 Hoagland nutrient solution (pH 5.95) for hydroponic cultivation. Tissue‐specific expression analysis was performed on roots, stems, and leaves collected from 4‐week‐old plants. For stress treatments, plants at the same developmental stage were subjected to drought stress (15% PEG6000) and leaf samples were collected at 0, 3, 5, 8, 12, 24, 48 and 72 h post‐treatment, with untreated plants serving as corresponding controls.

### 4.9 Alfalfa Phenotypic Analysis Under Drought Stress

Different alfalfa genotypes (wild‐type WT, overexpression and mutant lines) were vegetatively propagated, and uniform cuttings were selected for three parallel drought experiments.

Experiment 1: Controlled drought stress in growth chambers. Cuttings were transplanted into individual pots (8.5 × 8.5 × 9.5 cm) containing a pre‐weighed, standardized growth substrate (150 ± 5 g; vermiculite: nutrient soil = 2:1). To ensure uniform initial soil moisture, all pots were saturated by placing them in a tray filled with ~2 L of water overnight. After draining excess water, pots were transferred to controlled growth chambers. Drought stress was initiated by complete water withholding.

Experiment 2: Drought stress under near‐natural conditions in a greenhouse. Cuttings were transplanted into individual pots (11.5 cm height × 13 cm diameter) containing a pre‐weighed amount of sandy loam soil (960 ± 5 g) collected from the experimental site. The soil was pre‐dried, and each pot received an initial uniform watering of 300 mL. Pots were placed in a multi‐span greenhouse, and drought stress was initiated by withholding further irrigation.

Experiment 3: Drought was simulated using mannitol‐induced osmotic stress. Uniform cuttings were transferred to a hydroponic system containing 1/2 Hoagland nutrient solution and grown for 2 weeks. Plants with consistent growth vigour, size, and developmental stage across different genotypes were then selected for mannitol treatment.

To minimize micro‐environmental variation, the positions of all pots in both the growth chambers and the greenhouse were systematically randomized and re‐randomized on a weekly basis throughout the experiment. Stress severity was monitored by observing progressive phenotypic symptoms (e.g., leaf wilting). Phenotypic key parameters were recorded at the termination of stress.

### 4.10 Phenotypic Analysis of Arabidopsis Under Drought Stress

Drought tolerance of Arabidopsis was evaluated using the following two experimental systems:

Experiment 1: Seed germination assay under mannitol‐induced osmotic stress. Surface‐sterilized and cold‐stratified seeds of wild‐type, mutant, and transgenic Arabidopsis lines were sown on 1/2 MS solid media containing different concentrations of mannitol (0, 200, 250 and 300 mM), with three biological replicates per concentration. Plates were placed under light conditions, and the number of germinated seeds was recorded daily to calculate germination rates.

Experiment 2: Soil‐based drought simulation with mannitol treatment. Seeds of different lines were sterilized, stratified and grown on 1/2 MS solid medium for 8 days. Uniform seedlings were then transplanted into a standardized soil mixture (2:1 vermiculite:nutrient soil). After 2 weeks of growth, drought was simulated by applying a 50 mM mannitol solution for 5 days followed by 100 mM for 2 days quantitatively (10 mL) to each pot; control plants received an equal volume of water. Phenotypes were monitored and recorded throughout the treatment period. Similarly, all pots were systematically randomized and re‐randomized on a weekly basis throughout the experiment.

### 4.11 Determination of Relative Water Content (RWC) in Leaves

About 0.1 g fresh leaves were excised and immediately weighed to obtain the fresh weight (FW). Samples were immersed in 10 mL of deionized water in 15 mL conical tubes for 6 h at 25°C under dim light to achieve full turgidity. After surface moisture removal by filter paper blotting, turgid weight (TW) was recorded. Samples were then dehydrated in a forced‐air oven at 65°C for 72 h until constant dry weight (DW) was obtained. RWC was calculated using the formula:

$$\mathrm{RWC}\;\left(\%\right)=\left(\mathrm{FW}-\mathrm{DW}\right)/\left(\mathrm{TW}-\mathrm{DW}\right)\times 100.$$

### 4.12 Electric Conductivity Measurement

About 0.1 g of fresh leaves were immersed in 14 mL of ddH_2_O within 15 mL polypropylene centrifuge tubes. Samples were equilibrated on an orbital shaker (70 rpm) at 25°C for 24 h before initial conductivity measurement (R1). Control measurements (R0) were obtained from sterile ddH_2_O blanks. Following thermal treatment (100°C water bath for 30 min) and cooling to ambient temperature, maximum electrolyte conductivity (R2) was determined with corresponding blank controls (R0′). Electric conductivity was calculated as:

$$\text{Conductivity}\;\left(\%\right)=\left(\text{R1}-\text{R0}\right)/\left(\text{R2}-{\text{R0}}^{\prime }\right)\times 100.$$

### 4.13 Measurement of Photosynthetic Parameters

Leaf net photosynthetic rate (Pn), stomatal conductance (Gsw) and transpiration rate (Tr) were measured using an LI‐6800 portable photosynthesis system (LI‐COR, USA). Water use efficiency (WUE) was calculated as the ratio of Pn to Tr. The relative chlorophyll content of plant leaves was determined as SPAD (Soil and Plant Analyser Development) values using a portable chlorophyll meter (SPAD‐502, Konica Minolta, Japan).

### 4.14 Electrophoretic Mobility Shift Assay (EMSA)

The EMSA was performed using the LightShift Chemiluminescent EMSA Kit (Thermo Scientific) according to the manufacturer's instructions. Since the full‐length MsCBF8 protein could not be successfully expressed and purified, we generated a truncated form (lacking residues 201‐279) that was amenable to purification. Purified His‐MsCBF8 protein was incubated with a biotin‐labelled DNA probe derived from the *MsGolS2* promoter region, which contains the GCCGAC *cis*‐element. For competition assays, a 20‐ or 50‐fold unlabeled cold probe (TAGTGTTTTTTACGTGCCGACATGATAATAATATCA) was incubated with the biotin‐labelled DNA probe and purified His‐MsCBF8 protein. In addition, a corresponding mutated probe (TAGTGTTTTTTACGTaaaaAaATGATAATAATATCA) was incubated with His‐MsCBF8 protein to observe the binding capacity. Protein‐DNA complexes were resolved on a non‐denaturing polyacrylamide gel, transferred to a nylon membrane and detected using an ultrasensitive chemiluminescence imaging system (ChemiScope 6100, Shanghai, China).

### 4.15 Tobacco Dual‐Luciferase Assay for In Vivo Imaging and Dual‐Luciferase Reporter Assay

The *MsCBF8* was cloned into the Super1300 vector, while the promoter region of the *MsGolS2* was inserted into the pGreenII 0800‐LUC vector. Both constructs were individually transformed into *Agrobacterium tumefaciens* strain GV3101 (harbouring the pSoup‐p19 plasmid). Transformed *Agrobacterium* cultures were activated by growing in appropriate medium. Bacterial suspensions were adjusted to an optical density at 600 nm (OD₆₀₀) of 1.0 using an infiltration buffer. The infiltration buffer consisted of 500 mM MES (2 mL per 100 mL buffer), 100 mM Acetosyringone (As, 0.15 mL per 100 mL buffer), 100 mM MgCl_2_ (10 mL per 100 mL buffer) and deionized water (to make up the final volume). Equal volumes of the *Agrobacterium* suspensions carrying the effector and reporter constructs were mixed and incubated in the dark for 2 h at room temperature. The mixed suspension was then infiltrated into the leaves of tobacco (*Nicotiana benthamiana*) using a needleless syringe. Infiltrated areas were clearly marked. After 48 h of incubation under standard plant growth conditions, the leaves were used for fluorescence observation. Prior to imaging, the detached tobacco leaves were evenly sprayed with a working solution of D‐luciferin (150 μg/mL) until the leaf surface was fully moistened but not dripping. The leaves were subsequently dark‐adapted for 5 min. Thereafter, the leaves were placed in a low‐light cooled CCD camera system, and the luminescent signal was captured using an exposure time of 1–2 min. For dual‐luciferase assays, after 48 h of incubation under standard plant growth conditions, leaf discs were excised from the marked infiltration sites using a cork borer. Moreover, firefly luciferase (LUC) and Renilla luciferase (REN) activities were quantified using the Dual‐Luciferase Reporter Assay System (Promega) according to the manufacturer's instructions. The relative promoter activity was expressed as the ratio of LUC luminescence to REN luminescence (LUC/REN).

### 4.16 Virus‐Induced Gene Silencing (VIGS) in Alfalfa

A gene‐specific fragment of *MsGolS2* was designed using the VIGS online tool (https://vigs.solgenomics.net/). A partial coding sequence (~300 bp) was amplified and cloned into the pTRV2 vector. The confirmed recombinant plasmid was transformed into *Agrobacterium tumefaciens* strain EHA105, with the empty pTRV2 vector serving as the control. Virus‐induced gene silencing (VIGS) was performed by vacuum‐infiltration of alfalfa seedlings, following a previously described protocol (Liu, Shi, et al. 2023). After infiltration, plants were grown in the greenhouse for 5 days before assessing gene expression. Lines showing significant reduction in *MsGolS2* transcript levels were selected for subsequent drought treatment experiments.

### 4.17 Determination of Malondialdehyde (MDA) and Hydrogen Peroxide (H_2_O_2_ ) Contents and Superoxide Dismutase (SOD) and Catalase (CAT) Activities

Physiological indices including MDA content, H_2_O_2_ content, CAT activity and SOD activity were quantified using commercial assay kits (Nanjing Jiancheng Bioengineering Institute, China) in strict accordance with the manufacturer's protocols.

### 4.18 Leaf Stomatal Observation

The abaxial epidermis was carefully peeled using fine‐tipped forceps and immediately mounted in distilled water on glass slides. Stomatal architecture was examined under a light microscope (Olympus IX73) equipped with a digital camera (DP27). Per sample was captured at a 40× objective lens under standardized bright‐field illumination. All imaging procedures were conducted within 10 min of epidermal isolation to maintain cellular integrity, with 10 biological replicates analysed per genotype.

### 4.19 Statistical Analysis

Statistical analyses were performed using one‐way ANOVA followed by Duncan's multiple range test for post hoc comparisons (SPSS 26.0, IBM Corp.). Significant differences between treatment groups were determined at the *p* < 0.05 level. In addition to conventional significance testing, effect sizes (Cohen's *d* for two‐group comparisons) along with their 95% confidence intervals were calculated by independent sample *t*‐test to estimate the magnitude and precision of observed effects (see Data S1).

## Author Contributions

Y.Z., X.W. and J.K. designed the experiment; Y.Z., X.Q.J., T.Z., H.L., J.C., X.J. and L.Z. performed experiments; Y.Z., X.Q.J., Z.W., T.J.Z., Q.Y., X.W. and J.K. analysed the data; Y.Z. wrote the manuscript.

## Funding

This work was financially supported by the National Key Research and Development Program of China (2022YFF1003203), the Agricultural Science and Technology Innovation Program (ASTIP‐IAS14), and the Postdoctoral Fellowship Program of CPSF under Grant Number GZC20250161.

## Conflicts of Interest

The authors declare no conflicts of interest.

## Supporting information


**Figure S1:** Bioinformatics analysis of the MsCBF8 protein.
**Figure S2:** Tissue‐specific expression profiling of *MsCBF8*.
**Figure S3:** Molecular characterization of *MsCBF8* overexpression lines.
**Figure S4:** Survival rate of *MsCBF8*‐overexpressing transgenic lines and wild‐type (WT) controls following a one‐week recovery period post drought‐rewatering treatment.
**Figure S5:** Sanger sequencing of PCR amplicons (a) and single colonies (b) confirmed CRISPR/Cas9‐induced mutations at target Site 1 (ATCACAAAGATCCACCAACT) in the *mscbf8‐58* and *mscbf8‐59* mutant lines.
**Figure S6:** Mutation verification and expression analysis of *MsCBF8* edited lines.
**Figure S7:** Survival rate of mutations *mscbf8* transgenic lines and wild‐type (WT) controls following a one‐week recovery period post drought‐rewatering treatment.
**Figure S8:** Physiological analysis of drought tolerance in Arabidopsis following *MsCBF8* complementation.
**Figure S9:** MsCBF8 positively regulates freezing tolerance in alfalfa.
**Figure S10:** Phenotypic and physiological comparison between wild‐type alfalfa and empty vector control (Super1300) under normal and drought conditions.
**Table S1:** List of primer pairs used in this study.
**Table S2:** List of significant *k*‐mer markers used in this study.

**Data S1:** Complete dataset with significance testing and effect sizes (Cohen's *d* with 95% CI) for all two‐group comparisons in the study.

## Data Availability

Experimental datasets supporting the conclusions of this study are available in Appendix S1 and Data S1 of this article.
