# Supplementary material for: Converging GWAS and Genome Editing Decrypts MsCBF8‐ MsGolS2 Regulatory Module for Drought Tolerance Without Yield Penalty in Medicago sativa
Source: Plant Biotechnol J. 2026 Sep 22;24(11):5967–84. doi: 10.1111/pbi.70756 (PMC13596021; doi:10.1111/pbi.70756)
Supplement: Supplementary file 1 [file PBI-24-5967-s002.zip › pbi70756-sup-0001-AppendixS1/1 Revised-supporting information (clean).docx]

**Converging GWAS and genome editing decrypts MsCBF8-*MsGolS2* regulatory module for drought tolerance without yield penalty in *Medicago sativa***

Yangyang Zhang^1^, Xueqian Jiang^1^, Tian Zhang^1^, Huajuan Leng^1^, Jing Cui^1^, Xu Jiang^1^, Lili Zhang^1^, Zhen Wang^2^, Tiejun Zhang^1^, Qingchuan Yang^1^, Xue Wang^1,*^, Junmei Kang^1,*^

*^1^Institute of Animal Science, Chinese Academy of Agricultural Sciences, Beijing, 100000, China*

*^2^Department of Agronomy and Horticulture, University of Nebraska-Lincoln, Lincoln, NE 68583, USA*

**Corresponding authors. E-mail addresses: Xue Wang,* [*wangxue01@caas.cn*](mailto:wangxue01@caas.cn)*; Junmei Kang,* [*kangjunmei@caas.cn*](mailto:kangjunmei@caas.cn)

Yangyang Zhang: [yang20170115@163.com](mailto:yang20170115@163.com)

Xueqian Jiang: xueq_jiang@163.com

Tian Zhang: zt150944@163.com

Huajuan Leng: 13355322486@163.com

Jing Cui: [cuijing0417@yeah.net](mailto:cuijing0417@yeah.net)

Xu Jiang: jiangxu2009@yeah.net

Lili Zhang: lili_chang721@yeah.net

Zhen Wang: zwunl3@gmail.com

Tiejun Zhang: zhangtiejun@caas.cn

Qingchuan Yang: [yangqingchuan@caas.cn](mailto:yangqingchuan@caas.cn)

Xue Wang: wangxue01@caas.cn

Junmei Kang: [kangjunmei@caas.cn](mailto:kangjunmei@caas.cn)

**Figure S1** Bioinformatics analysis of the MsCBF8 protein. (a) Signal peptide prediction. (b) Secondary structure prediction. (c) Conserved domain analysis.

**Figure S2** Tissue-specific expression profiling of *MsCBF8*. Error bars represent the mean ± standard error of the mean (SEM) from three biological replicates. Statistical significance between groups was determined by one-way analysis of variance (ANOVA). Bars labeled with different uppercase letters indicate statistically significant differences at *P*<0.05 based on Duncan’s multiple range test.

**Figure S3** Molecular characterization of *MsCBF8* overexpression lines. (a) PCR validation of *MsCBF8* transgene integration. (b) qRT-PCR analysis of *MsCBF8* transcript levels in transgenic lines. Red arrows indicate the two lines selected for in detail analysis. Error bars represent the mean ± standard error of the mean (SEM) from three biological replicates. Statistical significance between groups was determined by one-way analysis of variance (ANOVA).

**Figure S4** Survival rate of *MsCBF8*-overexpressing transgenic lines and wild-type (WT) controls following a one-week recovery period post drought-rewatering treatment.

**Figure S5** Sanger sequencing of PCR amplicons (a) and single colonies (b) confirmed CRISPR/Cas9-induced mutations at target site 1 (ATCACAAAGATCCACCAACT) in the *mscbf8-58* and *mscbf8-59* mutant lines.

**Figure S6** Mutation verification and expression analysis of *MsCBF8* edited lines. (a) Sanger sequencing of single colonies confirmed the CRISPR/Cas9-induced mutation at target site 1 (ATCACAAAGATCCACCAACT) in the *mscbf8-9* mutant line. (b) Expression analysis of CRISPR-derived edited lines reveals significant changes in *MsCBF8* transcript levels. Error bars represent the mean ± standard error of the mean (SEM) from nine biological replicates. Statistical significance between groups was determined by one-way analysis of variance (ANOVA). Bars labeled with different lowercase letters indicate statistically significant differences at *P*<0.05 based on Duncan’s multiple range test.

**Figure S7** Survival rate of mutations *mscbf8* transgenic lines and wild-type (WT) controls following a one-week recovery period post drought-rewatering treatment.

**Figure S8** Physiological analysis of drought tolerance in Arabidopsis following *MsCBF8* complementation. (a-g) Changes of chlorophyll a content, chlorophyll b content, total chlorophyll content, malondialdehyde (MDA) content, hydrogen peroxide (H₂O₂) content, superoxide dismutase (SOD) activity, and catalase (CAT) activity in leaves of Arabidopsis mutant, wild-type, and two complementation lines (COM11, COM12) after drought stress treatment. Error bars represent the mean ± standard error of the mean (SEM) from three biological replicates. Statistical significance between groups was determined by one-way analysis of variance (ANOVA). Bars labeled with different lowercase letters indicate statistically significant differences at *P*<0.05 based on Duncan’s multiple range test.

**Figure S9** MsCBF8 positively regulates freezing tolerance in alfalfa. (a) Temporal changes in the transcript level of *MsCBF8* under cold stress (4°C). (b) Growth phenotypes of different alfalfa genotypes (*mscbf8* mutant, WT, and *MsCBF8*-overexpressing line *MsCBF8*-OE) under normal conditions and after freezing treatment. Prior to freezing treatment, 4-week-old plants were cold-acclimated at 4°C for one week. For the treatment, the temperature was gradually decreased at a rate of 1°C per hour until reaching -9°C, held for 1 hour, then transferred to 4°C overnight, followed by a 7-day recovery period in the greenhouse. (c) Changes in relative electrolyte leakage in leaves of different genotypes after freezing treatment. (d) Changes in relative chlorophyll content in leaves of different genotypes after freezing treatment. Error bars represent the mean ± standard error of the mean (SEM) from three biological replicates. Statistical significance between groups was determined by one-way analysis of variance (ANOVA). Bars labeled with different lowercase letters indicate statistically significant differences at *P*<0.05 based on Duncan’s multiple range test.

**Figure S10** Phenotypic and physiological comparison between wild-type alfalfa and empty vector control (Super1300) under normal and drought conditions. (a) Growth phenotypes of two-week-old hydroponically grown wild-type and empty vector control (Super1300) plants under normal conditions. (b) Phenotypic changes of the same plants after simulated drought treatment with 100 mM mannitol for 2 days. (c-j) Comparison of leaf physiological parameters between wild-type and empty vector control plants under normal and drought-stressed conditions: (c) net photosynthetic rate (Pn), (d) transpiration rate (Tr), (e) stomatal conductance (Gsw), (f) water use efficiency (WUE), (g) malondialdehyde (MDA) content, (h) hydrogen peroxide (H₂O₂) content, (i) superoxide dismutase (SOD) activity, and (j) catalase (CAT) activity. Error bars represent the mean ± standard error of the mean (SEM) from at least three biological replicates. Statistical significance between groups was determined by one-way analysis of variance (ANOVA). Bars labeled with different lowercase letters indicate statistically significant differences at *P*<0.05 based on Duncan’s multiple range test.

**Table S1** List of primer pairs used in this study.

| **Primer** | | **Sequence (5'-3')** |
| --- | --- | --- |
| **Primers amplified for MsCBF8-mutant constructs** | | |
| MsCBF8-2.0-F1 | | TAGGTCTCAATCGAACAAAGCACCAGTG |
| MsCBF8-2.0-R1 | | GCGGTCTCAGATCTTTGTGATTGCACCAGCCGGGAA |
| MsCBF8-2.0-F2 | | TAGGTCTCAGATCCACCAACTGTTTTAGAGCTAGAAA |
| MsCBF8-2.0-R2 | | GCGGTCTCAATCTCCGAATCCTGCACCAGCCGGGAA |
| MsCBF8-2.0-F3 | | TAGGTCTCAAGATACGATTAGGTTTTAGAGCTAGAA |
| MsCBF8-2.0-R3 | | TAGGTCTCAATATAAAAAAAGCACCGACTCGGTGCC |
| JC-2.0-F | | catcgaccggaagaggtaca |
| JC-2.0-R | | acgccagctggcgaaagggg |
| **Primers amplified for genomic PCR to detect gene editing plants** | | |
| MsCBF8-Cas9-JC-F | | TACAATAACTATAAAAGGAAGG |
| MsCBF8-Cas9-JC-R | | TGCCATATCGGCTGTTGGAAAA |
| **Primers amplified for MsCBF8-OE constructs** | | |
| MsCBF8-F | | ATGTTTACTA CTAACAACTC TTCCTATTC |
| MsCBF8-R | | TTATCCTTGT GGTAATTTCA GAATG |
| MsCBF8-1300-F | | tctagaaagcttctgcaggggATGTTTACTACTAACAACTC TTCCTATTC |
| MsCBF8-1300-R | | ctcctcgcccttgctcaccatTCCTTGTGGTAATTTCAGAATG |
| Super1300-F | | ACAACGCTTTACAGCAAGAACGG |
| Super1300-R | | GGTCAGGGTGGTCACGAGGG |
| **Primers for qRT-PCR detection** | | |
| MsACTIN-RT-F | CAAAAGATGGCAGATGCTGAGGAT | |
| MsACTIN-RT-R | CATGACACCAGTATGACGAGGTCG | |
| AtACTIN-RT-F | GAAATCACAGCACTTGCACC | |
| AtACTIN-RT-R | AAGCCTTTGATCTTGAGAGC | |
| MsCBF8-RT-F | GAGTTCCTTGGCCGCATTTC | |
| MsCBF8-RT-R | TCTTTTGCACCTTCCCTTGCTA | |
| MsGolS2-RT-F | CGTCACACCAAGCAGTATGAAA | |
| MsGolS2-RT-R | CTCAGCAAAGGAAGTTGGCTC | |
| **Other primers** | | |
| MsGolS2-pTRV2-F | ctctagaaggcctccatggg ACCAACACTC AATCTAAGGC | |
| MsGolS2-pTRV2-R | cgtgagctcggtaccggatc ATAGTCCTCA AAAGCCCAAA | |
| MsGolS2-LUC-F | tgatatcgaattcctgcagcccTGTACAATTC TAAATCCCAC | |
| MsGolS2-LUC-R | tggcggccgctctagaactagtAATACTTGGC AACCATATCA | |
| MsCBF8-PGBKT7-F | CATGGAGGCCGAATTCATGTTTACTACTAACAACTCTTCCTATTCA | |
| MsCBF8-PGBKT7-R | GCAGGTCGACGGATCC TTATCCTTGT GGTAATTTCAGAATG | |

**Table S2** List of significant k-mer markers used in this study.

| **k-mer** | **Chr.** | **Pos.** | ***P*** | **-log10(*P*)** |
| --- | --- | --- | --- | --- |
| kmer_chr1_23325081 | chr1 | 23325081 | 3.41E-09 | 8.466906087 |
| kmer_chr1_66549407 | chr1 | 66549407 | 1.31E-09 | 8.883944107 |
| kmer_chr1_70791866 | chr1 | 70791866 | 2.36E-08 | 7.626568075 |
| kmer_chr1_75207858 | chr1 | 75207858 | 4.06E-08 | 7.390943617 |
| kmer_chr1_78594253 | chr1 | 78594253 | 1.60E-08 | 7.795342097 |
| kmer_chr2_9260696 | chr2 | 9260696 | 6.69E-08 | 7.174615561 |
| kmer_chr2_38030438 | chr2 | 38030438 | 4.25E-09 | 8.37146701 |
| kmer_chr2_49621697 | chr2 | 49621697 | 2.50E-09 | 8.602147206 |
| kmer_chr2_55367850 | chr2 | 55367850 | 9.56E-08 | 7.019668917 |
| kmer_chr2_62782160 | chr2 | 62782160 | 2.75E-08 | 7.561259298 |
| kmer_chr2_66522258 | chr2 | 66522258 | 8.51E-08 | 7.070171089 |
| kmer_chr2_78934848 | chr2 | 78934848 | 2.05E-08 | 7.687914295 |
| kmer_chr3_25012474 | chr3 | 25012474 | 8.54E-08 | 7.068591613 |
| kmer_chr3_65878853 | chr3 | 65878853 | 7.39E-08 | 7.13155877 |
| kmer_chr3_66719472 | chr3 | 66719472 | 2.27E-08 | 7.643692612 |
| kmer_chr3_72036729 | chr3 | 72036729 | 2.55E-08 | 7.594125055 |
| kmer_chr3_74488791 | chr3 | 74488791 | 1.31E-08 | 7.881203399 |
| kmer_chr3_75004520 | chr3 | 75004520 | 1.13E-08 | 7.948624404 |
| kmer_chr4_14614486 | chr4 | 14614486 | 7.78E-08 | 7.10926944 |
| kmer_chr4_39806150 | chr4 | 39806150 | 7.88E-08 | 7.10320728 |
| kmer_chr4_57742198 | chr4 | 57742198 | 5.59E-09 | 8.252448914 |
| kmer_chr4_63657641 | chr4 | 63657641 | 2.20E-08 | 7.657893877 |
| kmer_chr4_65035271 | chr4 | 65035271 | 4.44E-08 | 7.352171324 |
| kmer_chr4_74678212 | chr4 | 74678212 | 3.54E-08 | 7.451570656 |
| kmer_chr4_77286597 | chr4 | 77286597 | 6.41E-09 | 8.193244357 |
| kmer_chr6_5800212 | chr6 | 5800212 | 6.75E-08 | 7.170554702 |
| kmer_chr6_8468285 | chr6 | 8468285 | 8.71E-08 | 7.059745814 |
| kmer_chr6_30953719 | chr6 | 30953719 | 5.65E-08 | 7.247881072 |
| kmer_chr6_45934972 | chr6 | 45934972 | 5.51E-08 | 7.258884896 |
| kmer_chr6_57431027 | chr6 | 57431027 | 6.19E-08 | 7.208052919 |
| kmer_chr6_69525041 | chr6 | 69525041 | 4.25E-08 | 7.371153616 |
| kmer_chr7_13089540 | chr7 | 13089540 | 3.61E-08 | 7.442235906 |
| kmer_chr7_26649629 | chr7 | 26649629 | 5.42E-08 | 7.265875491 |
| kmer_chr7_46334520 | chr7 | 46334520 | 7.09E-08 | 7.14932663 |
| kmer_chr7_59516748 | chr7 | 59516748 | 9.45E-08 | 7.024605143 |
| kmer_chr7_74593339 | chr7 | 74593339 | 9.73E-08 | 7.012055331 |
| kmer_chr7_78109554 | chr7 | 78109554 | 8.58E-09 | 8.066515952 |
| kmer_chr8_1158425 | chr8 | 1158425 | 8.80E-08 | 7.055626853 |
| kmer_chr8_12754674 | chr8 | 12754674 | 6.99E-08 | 7.155353489 |
| kmer_chr8_20655296 | chr8 | 20655296 | 9.74E-08 | 7.011421335 |
| kmer_chr8_39177681 | chr8 | 39177681 | 4.49E-09 | 8.347966215 |
| kmer_chr8_44494491 | chr8 | 44494491 | 2.01E-08 | 7.69638994 |
| kmer_chr8_46646148 | chr8 | 46646148 | 3.86E-08 | 7.413827498 |
| kmer_chr8_59493818 | chr8 | 59493818 | 1.18E-08 | 7.929397733 |
| kmer_chr8_69853240 | chr8 | 69853240 | 9.50E-08 | 7.022494191 |
| kmer_chr8_73398954 | chr8 | 73398954 | 9.26E-09 | 8.033490095 |
| kmer_chr8_86070171 | chr8 | 86070171 | 4.49E-08 | 7.347333979 |
| kmer_chr8_87388578 | chr8 | 87388578 | 2.75E-08 | 7.560170915 |
